# An Endohyphal Bacterium (*Chitinophaga*, Bacteroidetes) Alters Carbon Source Use by *Fusarium keratoplasticum* (*F. solani* Species Complex, Nectriaceae)

**DOI:** 10.3389/fmicb.2017.00350

**Published:** 2017-03-14

**Authors:** Justin P. Shaffer, Jana M. U'Ren, Rachel E. Gallery, David A. Baltrus, A. Elizabeth Arnold

**Affiliations:** ^1^School of Plant Sciences, University of ArizonaTucson, AZ, USA; ^2^Department of Agricultural and Biosystems Engineering, University of ArizonaTucson, AZ, USA; ^3^School of Natural Resources and the Environment, University of ArizonaTucson, AZ, USA; ^4^Department of Ecology and Evolutionary Biology, University of ArizonaTucson, AZ, USA

**Keywords:** endobacteria, fusaria, Gram-negative, phenotypic microarray, substrate use, symbiosis

## Abstract

Bacterial endosymbionts occur in diverse fungi, including members of many lineages of Ascomycota that inhabit living plants. These endosymbiotic bacteria (endohyphal bacteria, EHB) often can be removed from living fungi by antibiotic treatment, providing an opportunity to assess their effects on functional traits of their fungal hosts. We examined the effects of an endohyphal bacterium (*Chitinophaga* sp., Bacteroidetes) on substrate use by its host, a seed-associated strain of the fungus *Fusarium keratoplasticum*, by comparing growth between naturally infected and cured fungal strains across 95 carbon sources with a Biolog® phenotypic microarray. Across the majority of substrates (62%), the strain harboring the bacterium significantly outperformed the cured strain as measured by respiration and hyphal density. These substrates included many that are important for plant- and seed-fungus interactions, such as D-trehalose, *myo*-inositol, and sucrose, highlighting the potential influence of EHB on the breadth and efficiency of substrate use by an important *Fusarium* species. Cases in which the cured strain outperformed the strain harboring the bacterium were observed in only 5% of substrates. We propose that additive or synergistic substrate use by the fungus-bacterium pair enhances fungal growth in this association. More generally, alteration of the breadth or efficiency of substrate use by dispensable EHB may change fungal niches in short timeframes, potentially shaping fungal ecology and the outcomes of fungal-host interactions.

## Introduction

Plant-fungus interactions shape plant health and productivity in all terrestrial ecosystems (Heilmann-Clausen and Boddy, 2008; Kivlin et al., 2011; Tedersoo et al., 2014; Davison et al., 2015). Pathogens can negatively influence photosynthesis, nutrient- and water uptake and transport, structural integrity, reproduction, and seed germination of their hosts (Blanchette, 1991; Agrios, 1997; Gallery et al., 2007; Grimmer et al., 2012; Oliva et al., 2014). In turn, mycorrhizal fungi and some endophytes may enhance nutrient uptake and growth, alter plant water relations, or deter antagonistic microbes or herbivores (Arnold et al., 2003; Waller et al., 2005; Arnold and Engelbrecht, 2007; Busby et al., 2015; Estrada et al., 2015; van der Heijden et al., 2015).

Outcomes of such interactions are influenced by genetic and environmental factors (Schafer and Kotanen, 2003; Gallery et al., 2010; see Agrios, 1997; Jones and Dangl, 2006), but also can be shaped by additional microorganisms that alter fungal phenotypes (Frey-Klett et al., 2007; Márquez et al., 2007). Such microbes may occur on the exterior surfaces or interior of fungal cells, where they can alter sporulation, substrate use, metabolite production, and other features relevant to fungal interactions with plants (Partida-Martínez and Hertweck, 2005; Salvioli et al., 2010; Hoffman et al., 2013; Spraker et al., 2016). In particular, many plant-associated fungi harbor endosymbiotic bacteria (hereafter, endohyphal bacteria, EHB) that can affect host function and subsequent plant-fungus interactions (Partida-Martínez and Hertweck, 2005; Hoffman et al., 2013; Salvioli et al., 2016). EHB are known among diverse fungi (Hoffman and Arnold, 2010; Desirò et al., 2015; Shaffer et al., 2016), but only a few have been developed as model systems in which their effects have been observed.

The majority of studies to date focus on EHB in diverse root-associated and soilborne fungi, particularly Mucoromycotina, Mortierellomycotina, and Glomeromycotina (Bianciotto et al., 2003; Bertaux et al., 2005; Partida-Martínez et al., 2007b; Sharma et al., 2008; Sato et al., 2010; Desirò et al., 2015). Many of these EHB influence the phenotype of their fungal hosts. For example, the bacterium *Burkholderia rhizoxinica* produces a virulence factor that enables its host fungus *Rhizopus microsporus* (Mucoromycotina) to cause disease on rice (Partida-Martínez and Hertweck, 2005; Partida-Martínez et al., 2007b). When the bacterium is removed, the fungus is no longer pathogenic and loses the ability to reproduce asexually (Partida-Martínez and Hertweck, 2005; Partida-Martínez et al., 2007a). The bacterium *Candidatus* Glomeribacter gigasporarum increases responsiveness to strigolactones exuded by roots, enhancing hyphal elongation and branching relevant to mycorrhizal establishment by *Gigaspora margarita* (Gigasporaceae, Glomeromycotina) (Bianciotto et al., 1996, 2003, 2004; Lumini et al., 2007; Anca et al., 2009).

EHB also occur in Basidiomycota, with case studies beginning to highlight the functional aspects of their associations in rhizosphere and phyllosphere fungi (Bertaux et al., 2005; Izumi et al., 2005; Sharma et al., 2008; Ruiz-Herrera et al., 2015). For example, *Rhizobium radiobacter* (syn. *Agrobacterium tumefaciens*), like its host, *Piriformospora indica* (Sebacinales), can promote growth and induce disease resistance in barley (Sharma et al., 2008). Similarly, a *Bacillus* sp. fixes and makes available atmospheric nitrogen within cells of *Ustilago maydis* (Ustilaginomycotina; Ruiz-Herrera et al., 2015).

EHB recently have been documented in diverse Ascomycota, including members of multiple classes (Pezizomycetes, Eurotiomycetes, Dothideomycetes, and Sordariomycetes) and multiple functional groups (Barbieri et al., 2000; Hoffman and Arnold, 2010; Shaffer et al., 2016). They are widespread in foliar endophytes and in soilborne Ascomycota (e.g., those that interact with seeds; Hoffman and Arnold, 2010; Shaffer et al., 2016). To date their functional significance has been assessed in detail in only one study system: the foliar endophyte *Pestalotiopsis* sp. (Amphisphaeriaceae, Xylariales, Sordariomycetes) and its EHB, *Luteibacter* sp. (Xanthomonadaceae, Gammaproteobacteria; Hoffman et al., 2013; Arendt, 2015). More generally, studies of EHB in the Ascomycota and other fungi have focused primarily on Proteobacteria, Firmicutes, and Mollicutes (e.g., Desirò et al., 2015; see also Baltrus et al., 2016), leaving gaps with regard to the potential for symbiotic modulation of fungal phenotypes by members of other bacterial lineages.

Here, we use a phenotypic microarray (PM) to explore the influence of an EHB on its fungal host under laboratory conditions. Approaches to evaluate phenotypic effects of EHB on host fungi range from predictive analyses based on genomics and related tools to assays that use infected and cured strains of the same fungus (Anca et al., 2009; Ghignone et al., 2012; Hoffman et al., 2013). The advantage of our approach is that it allows quantification of respiration and growth on 95 substrates simultaneously, providing within-experiment controls and comparisons that are not subject to variation introduced in a lower-throughput framework (Atanasova et al., 2010; Druzhinina et al., 2010; Blumenstein et al., 2015a,b). PMs have been used in diverse studies involving bacteria (reviewed in Bochner, 2008), and since their development have been extended to work with fungi, including yeasts and filamentous strains (reviewed in Bochner, 2003; Druzhinina et al., 2006; Atanasova and Druzhinina, 2010; Pfliegler et al., 2014). To our knowledge, PMs have not been used previously to explore interactions among microbes or more specifically, the effects of bacterial endosymbionts on fungal phenotypes.

In this study we focus on a lineage of bacteria that has not yet been evaluated for phenotypic modulation of fungi: the Bacteroidetes, a diverse clade of Gram-negative, non-endospore forming bacteria that are known from soils and from diverse symbiotic associations (Krieg et al., 2010; Thomas et al., 2011). Specifically we examine the effects of a strain of *Chitinophaga* sp. (Bacteroidetes) on substrate use by its host fungus, a strain of *Fusarium keratoplasticum* (*F. solani* species complex, FSSC, *sensu* Short et al., 2013). The fungal strain was originally isolated from the interior of a seed of a tropical tree that was retrieved from soil in a tropical forest. Subcultures of the strain consistently harbor *Chitinophaga* sp., and preliminary analyses indicated that this bacterium can be removed by antibiotic treatment. Here we show that this endohyphal *Chitinophaga* alters substrate use and substrate-specific growth by its host fungus. Our results highlight the importance of EHB with regard to shaping fungal phenotypes relevant to plant-fungus interactions and demonstrate the capacity of a focal member of the Bacteroidetes to alter the functional traits of a fungal species that includes medically and ecologically important strains.

## Materials and methods

As part of a previous study (Sarmiento et al., 2015; Zalamea et al., 2015), a fungus was isolated from a seed of *Cecropia insignis* (Urticaceae) that had been buried for 1 month in the soil in the tropical forest understory at Barro Colorado Island, Panama (9° 10′N, 79° 51′W; 86 m.a.s.l.; for a site description see Leigh, 1999). The seed was retrieved from the soil, washed in water, and surface-sterilized by sequential immersion in 95% ethanol (10 s), diluted chlorine bleach (0.525% NaClO; 2 min), and 70% ethanol (2 min) (Arnold and Lutzoni, 2007). The seed was cut in half and allowed to surface-dry under sterile conditions. One half of the seed was placed on 2% malt extract agar (MEA) following Gallery et al. (2007). Fungal isolate PS0362A, containing its bacterial symbiont, grew from the interior of the seed. PS0362A was deposited as a living voucher at the Robert L. Gilbertson Mycological Herbarium, University of Arizona.

Phylogenetic analyses based on three loci identified PS0362A as *F. keratoplasticum*, a member of the *Fusarium solani* species complex (FSSC) (Nectriaceae, Hypocreales, Sordariomycetes, Ascomycota; Shaffer et al., 2016). Its bacterial symbiont was observed during screening and was identified by phylogenetic analysis of the 16*S* ribosomal RNA (rRNA) gene as *Chitinophaga* sp. PS-EHB01 (Bacteroidetes) (Shaffer et al., 2016). We have refined the placement of *Chitinophaga* sp. PS-EHB01 within the Chitinophagaceae by focusing taxon sampling and inferring a new 16*S* phylogeny, confirming its taxonomic identity (see Supplementary Materials and Methods).

### Preparation of axenic fungal culture

*Fusarium keratoplasticum* PS0362A was naturally infected by *Chitinophaga* sp. PS-EHB01. We prepared an axenic strain of the fungus by subculturing hyphae under sterile conditions on 2% MEA amended with four antibiotics: ampicillin (100 μg/mL), kanamycin (50 μg/mL), tetracycline (10 μg/mL), and ciprofloxacin (40 μg/mL; see Hoffman and Arnold, 2010; Hoffman et al., 2013; Arendt et al., 2016). We refer to clones of the naturally infected fungus as the EHB+ strain, and those of the axenic fungus as the EHB− strain.

We confirmed the presence or absence of *Chitinophaga* sp. PS-EHB01 in *F. keratoplasticum* PS0362A through light microscopy, molecular analysis, and fluorescence microscopy. We first ruled out extrahyphal bacteria (i.e., contaminants in the medium or microbes on hyphal surfaces; see Supplementary Figure 1) by examining five samples of hyphae per culture at 400X and 1,000X on a Leica DM400B compound microscope. We did not observe extrahyphal bacteria in any cultures of the EHB+ or EHB− strains used here.

We extracted total genomic DNA from fresh mycelia of EHB+ and EHB− strains using the REDExtract-N-Amp Plant Kit (Sigma-Aldrich, St. Louis, MO, USA) following a modified protocol (see U'Ren, 2016; U'Ren et al., 2016). We prepared five separate DNA extractions per strain type. We used the polymerase chain reaction (PCR) to amplify a *ca*. 1,400 base pair (bp) segment of the 16*S* rRNA gene (forward primer 27F, reverse primer 1492R; 10 μM; Lane, 1991) with PCR cycling parameters following Hoffman and Arnold (2010) (3 min at 94°C, 40 cycles of 30 s at 94°C, 30 s at 55°C, and 2 min at 72°C, followed by 10 min at 72°C). For each reaction we used 10 μL of PCR Ready Mix, 0.8 μL of each primer, 4.4 μL of molecular grade water, and 4 μL of DNA template. We used molecular grade water instead of template in negative controls. Negative controls never resulted in visible, positive PCR products. Positive controls consisted of a bacterial strain that was amplified consistently with these primers. Positive controls yielded amplification as expected in every PCR.

Positive PCR products were cleaned by adding 1 μL ExoSAP-IT (Affymetrix, Santa Clara, CA, USA) to each of the remaining products. Reactions were incubated on a thermal cycler at 37°C for 60 min, and then at 80°C for 15 min to deactivate enzymes. Cleaned PCR products were diluted 1:1 with molecular grade water and sequenced bidirectionally on an AB3730XL (Applied Biosystems, Foster City, CA, USA) using PCR primers (5 μM) at the University of Arizona Genomics Core.

An assembly pipeline consisting of *phred* and *phrap* (Ewing and Green, 1998; Ewing et al., 1998) driven by *Chromaseq* (Maddison and Maddison, 2005) in Mesquite v.2.75 (Maddison and Maddison, 2009) was used to call bases and assemble bidirectional reads into contigs. Base calls were verified by manual inspection of all chromatograms in Sequencher v.5.1 (Gene Codes Corp., Ann Arbor, MI, USA).

We consistently found *Chitinophaga* sp. PS-EHB01 in cultures of the EHB+ strain of *F. keratoplasticum* PS0362A. No other bacteria were observed in that strain. *Chitinophaga* sp. PS-EHB01 was not detected in cultures of the EHB− strain of *F. keratoplasticum* PS0362A, and we observed no other bacteria in clones of that strain.

To confirm that *Chitinophaga* sp. PS-EHB01 was viable and that it occurred within viable hyphae, we examined living hyphae by microscopy with the Live/Dead BacLight Bacterial Viability Kit (Invitrogen, Carlsbad, CA, USA) following Hoffman and Arnold (2010). The kit provides a two-color fluorescence assay of bacterial viability that uses two dyes: SYTO 9, a green-fluorescent nucleic acid stain that labels all cells, and propidium iodide, a red-fluorescent nucleic acid stain that labels only those cells with damaged or otherwise compromised cell membranes (see manufacturer's instructions).

We prepared fungal cultures for Live/Dead visualization by removing a small piece of mycelium (≤2-mm^2^) from the growing edge of a single colony growing on 2% MEA. Fragments were aseptically transferred to glass slides containing 20 μL of 1:1:18 Live/Dead stain (component A: component B: molecular grade water), teased apart using sterile insect mounting needles (size 00; BioQuip, Rancho Dominguez, CA, USA), covered with a coverslip, and incubated in the dark for 15 min. After incubation, we washed the mycelium by pulling molecular grade water through the slide mounts with bibulous paper and sealed the slides with two coats of nail polish. We used a Leica DM400B compound microscope with a 100-W mercury arc lamp for fluorescent imaging. Samples were viewed at room temperature with a Chroma Technology 35002 filter set (480-nm excitation/520-nm emission) and 100X APO oil objective.

**Figure 1:**
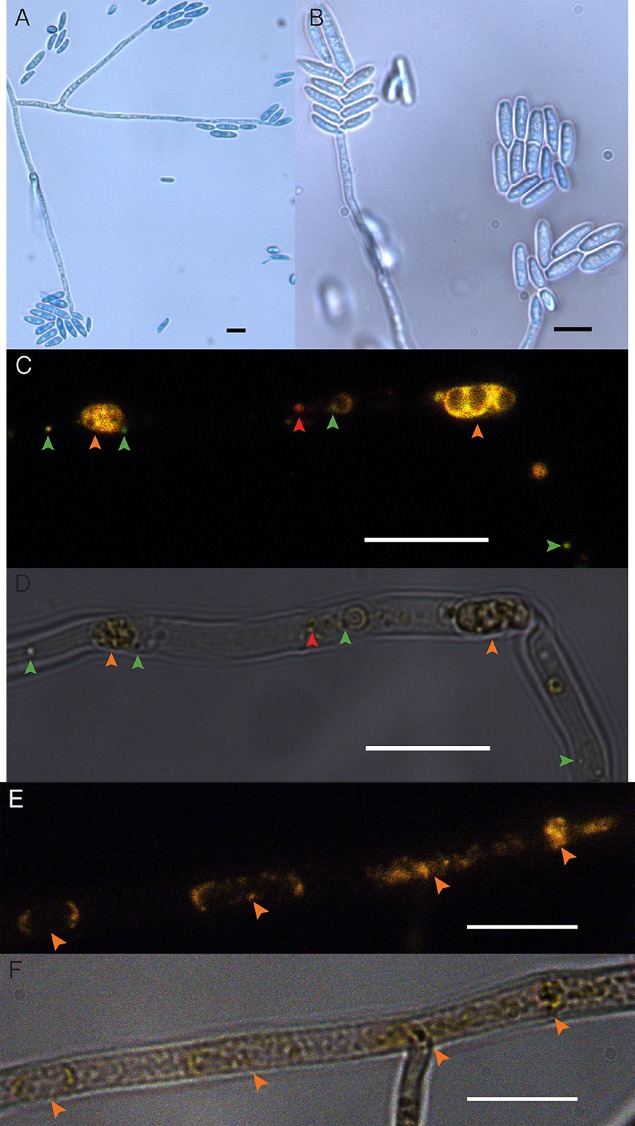
***Fusarium keratoplasticum* strain PS0362A. (A)** Details of culture on 2% MEA: hyphae, conidiophores, and macroconidia. **(B)** Conidiophores bearing macroconidia. **(C)** The EHB+ strain. Fluorescently tagged nucleic acids of viable bacteria appear green, those with damaged membranes in red, and compromised fungal organelles in orange. **(D)** Same frame as **(C)** viewed with differential interference contrast (DIC). In **(C,D)**, orange arrows indicate fungal nuclei, green arrows indicate viable EHB, and the red arrow indicates inviable EHB. **(E)** The cured strain (EHB−). Fluorescently tagged nucleic acids of compromised fungal organelles in orange. **(F)** Same frame as **(E)** viewed with DIC. In **(E,F)**, orange arrows indicate fungal nuclei. In all images fungal mycelium was alive at the outset of preparation but was inactivated during the visualization process. Scale bars = 10 μm.

Cultures of the EHB+ strain of *F. keratoplasticum* PS0362A consistently displayed fluorescence of nucleic acids distinct from fungal mitochondrial or nuclear DNA (Figure 1). Combined with the absence of extrahyphal bacteria and successful amplification of *Chitinophaga* sp. PS-EHB01 16*S* rRNA genes from genomic DNA isolated directly from the fungal culture, these results served as evidence of EHB+ status (Hoffman and Arnold, 2010; Arendt et al., 2016; Shaffer et al., 2016). Cultures of the EHB− strain did not contain visible fluorescence as above (Figure 1), and PCR amplification of bacterial 16*S* rRNA genes failed in these strains. The EHB+ and EHB− status of *F. keratoplasticum* PS0362A strains was confirmed before and after preliminary assays and Biolog® assays (below).

### Preliminary assays

We compared colony diameter and spore production between EHB+ and EHB− strains of *F. keratoplasticum* PS0362A by comparing five clones of each strain growing on 2% MEA. For each clone, we placed a 4-mm plug onto 2% MEA (15 mL) in a 100-mm Petri plate. Each plug was excised from just within the growing edge of a fresh culture growing for 5 days on 2% MEA in a 100-mm Petri plate. Plates were then incubated at 25°C for 10 d. At that point the colony diameter was *ca*. 5 mm from the edge of a 100-mm Petri plate, and aerial hyphae were numerous. To compare colony diameter, we marked the diameter of each clone across two orthogonal axes using a fine tip permanent marker, photographed each culture plate with aid from a tracing LED lightbox, and obtained the colony diameter for each clone by taking the average of the two axes as measured in ImageJ (Schneider et al., 2012). To compare spore production, we obtained spores from each clone by flooding the surface of the plate with 5 mL of sterilized milli-q H_2_O (sH_2_O), scraped the surface using a sterile rubber policeperson, and transferred the suspension to a sterile 50-mL Falcon tube (Corning, NY, USA). For each clone, we quantified the number of spores per mL of suspension using a hemocytometer.

### Biolog® assays

We used commercially available Biolog® microplates for phenotypic microarray assays. Biolog® filamentous fungus (FF) microplates (Catalog #1006, Biolog Inc., Hayward, CA, USA) are 96-well microtiter plates containing 95 unique carbon sources and one negative control (H_2_O) (Supplementary Table 1). Substrates and reagents are pre-filled and dried into wells. Redox chemistry based on the reduction of iodonitrotetrazolium violet (INT) produces a red-colored formazan dye with peak absorbance at 490 nm (Bochner and Savageau, 1977; Kubicek et al., 2003). This provides a colorimetric measure of mitochondrial activity resulting from substrate use (i.e., oxidation of succinate to fumarate in the citric acid cycle causes INT to be reduced; Bochner and Savageau, 1977; Bochner, 1989; Bochner et al., 2001; Kubicek et al., 2003). Reduction of INT and production of formazan cannot be reversed, and the quantitative measure of formazan accumulation by spectrophotometry reflects oxidation of the substrate in a particular well (Bochner, 1989; Bochner et al., 2001; Kubicek et al., 2003). In turn, reading the plates at 750 nm measures turbidity, which reflects growth of the fungus through substrate use and production of mycelium (Kubicek et al., 2003; Druzhinina et al., 2006; Atanasova and Druzhinina, 2010; Blumenstein et al., 2015b). Based on these measurements, the plates can distinguish even closely related strains within fungal species (Singh, 2009; Atanasova and Druzhinina, 2010).

Although FF microplates were designed for use with sporulating fungi, non-sporulating fungi can be evaluated on the plates following inoculation with homogenous hyphal suspensions (Singh, 2009). We assessed effects of EHB using hyphal suspensions for two reasons: first, the fungus produces conidia but we have not yet observed EHB in conidia of PS0362A following staining as described above (Supplementary Figure 2). Second, the fungus appears to colonize seeds as hyphae in natural conditions (Sarmiento et al., 2015).

We prepared inoculum by incubating mycelium from two clones of the EHB+ strain and two clones of the EHB− strain of *F. keratoplasticum* PS0362A on 2% MEA at 25°C for 10 d. At that point the colony diameter was *ca*. 5 mm from the edge of a 100-mm Petri plate, and aerial hyphae were numerous, as above. We flooded the surface of each plate with 5 mL of sH_2_O, scraped the surface using a sterile rubber policeperson, and combined suspension from both plates of the same EHB status by pouring into a sterile 50-mL Falcon tube. We separated and excluded conidia by filtering the suspensions through three layers of sterile cheesecloth, discarding the filtrate, and transferring trapped hyphae to new tubes. We brought the total volume of each tube up to 20 mL with 0.2% carrageenan type II media (see Hobbie et al., 2003). We then transferred each suspension to a sterile Waring blender cup and blended for 20 s. The suspension was allowed to cool for 20 s and then blended again for 20 s (see Gale et al., 1960; Orbach et al., 1996). We let each suspension rest for 10 min to allow large fragments to fall out of suspension, and diluted each with 0.2% carrageenan type II media to obtain an absorbance of 0.22 at 600 nm. We added 3 mL of this diluted suspension to 27 mL of 0.2% carrageenan type II media to produce the final hyphal suspension (Hobbie et al., 2003) (Supplementary Figure 3). We allowed suspensions to sit for 6 h at room temperature, confirming that any remaining conidia had germinated and produced at least one septum distal to the germ tube (Supplementary Figure 3B).

We inoculated 100 μL of suspension into each well of each microplate, pipetting carefully to avoid creating bubbles, and sealed each plate with a double layer of Parafilm (Bemis, Neenah, WI, USA). We prepared five replicate microplates for EHB+ and EHB− strains, wrapped all ten plates in aluminum foil, placed them into a plastic freezer bag with moistened paper towels to prevent drying, and incubated them at 25°C. Preliminary examination of hyphal suspension added to a synthetic glucose medium (20 mM) confirmed viability and growth of the inocula prepared as above.

We obtained data for substrate use by reading plates at 490 nm (absorbance corresponding to cellular respiration) and 750 nm (absorbance corresponding to hyphal density) every 12 h for 7 d. Plates were read using a Synergy H1 hybrid reader and accompanying Gen5 v.1.11 software package (BioTek, Winooski, VT, USA). We defined absorbance for a given strain on a given substrate on a given plate ($A_{\lambda }^{t}$) as the value read at a given wavelength (λ) at a specific time point during the experiment (t), and total absorbance by a given strain on a given substrate (${Ā}_{\lambda }^{t}$) as the mean absorbance from five replicate plates, measured at a given wavelength (λ) at a specified time point during the experiment (t). As previous studies have shown the absorbance spectrum of hyaline mycelium to be level over wavelengths from 490 to 750 nm, an adjusted redox value for the production of formazan is obtained by subtracting the absorbance for hyphal density (750 nm) from that for cellular respiration (490 nm; Tanzer et al., 2003; Atanasova and Druzhinina, 2010). We therefore define the corrected absorbance at 490 nm as *A*_c490_ = *A*_490_ − *A*_750_.

**Figure 2:**
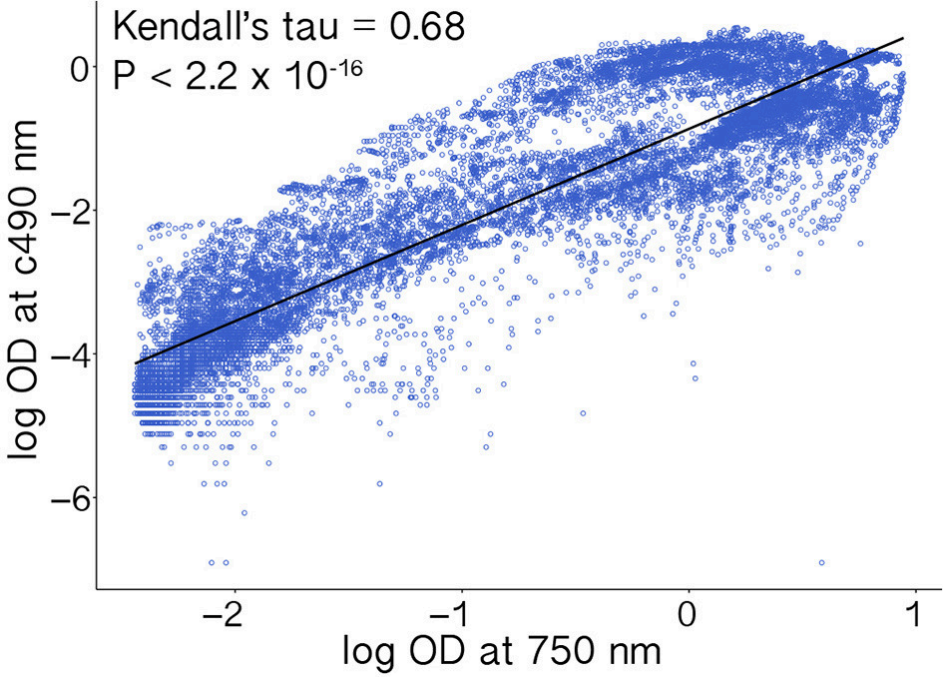
**Absorbance measurements corresponding to cellular respiration (*A*_c490_) and hyphal density (*A*_750_) were highly correlated**. Values were log transformed prior to analysis.

Absorbance measurements corresponding to respiration (*A*_c490_) and hyphal density (*A*_750_) were highly positively correlated (Kendall's τ = 0.68, *p* < 2.2 × 10^−16^; Figure 2). Furthermore, because similar studies may use indicator dyes other than INT (see Bochner and Savageau, 1977) and redox-based color formation by filamentous fungi does not always correlate with growth as with bacteria and yeasts (Atanasova and Druzhinina, 2010), absorbance values corresponding to hyphal density (750 nm) are more consistent across different growth conditions and hyphal morphologies. Absorbance values corresponding to hyphal density also are more often reported and therefore more comparable among studies (Tanzer et al., 2003; Atanasova and Druzhinina, 2010). Thus, we focus our results on measurements of absorbance (i.e., turbidity) corresponding to hyphal density (*A*_750_), which we refer to as growth (below).

**Figure 3:**
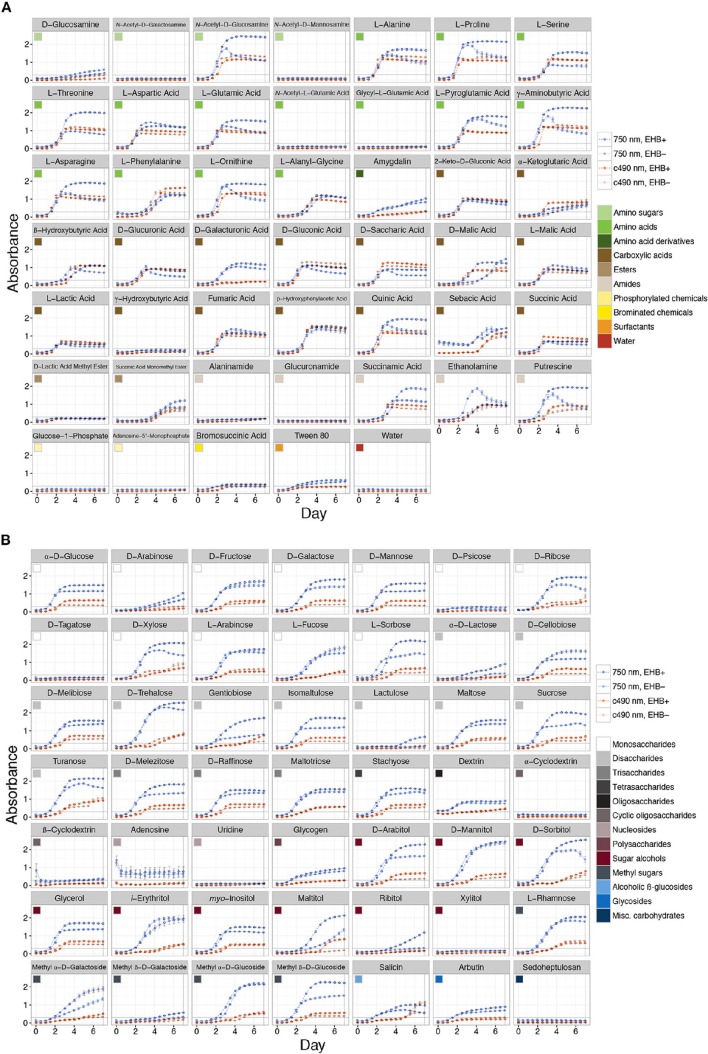
**Total absorbance for EHB+ and EHB− strains of *Fusarium keratoplasticum* PS0362A across all substrates on Biolog® phenotypic microarrays over 7 d**. Light- and dark-orange lines represent absorbance at 490 nm (i.e., cellular respiration). Light- and dark-blue lines represent absorbance at 750 nm (i.e., hyphal density, defined here as growth). Dark, dotted lines with open circles indicate absorbance for EHB+ strains. Light, solid lines with filled circles indicate absorbance for EHB− strains. Colored squares indicate different substrate classes. The horizontal line at Absorbance = 0.3 indicates the minimum absorbance value recognized. The vertical line at Day = 7 indicates the time point for which absorbance values were formally compared. **(A)** Sugar-based substrates. **(B)** Amino- and carboxylic acids, their derivatives, and other substrates.

Measurable growth was defined as 0.3 < $A_{750}^{7}$ ≤ 3.0 (see Figure 3), where the upper bound represents 99.9% light absorbance and the lower bound the value that best differentiates growth in the lag phase (i.e., negligible growth) from that reaching the log/exponential phase, across all substrates. We recognized absorbance values at which strains reach stationary phase as representing the maximum capacity for growth on that substrate. We observed a range of growth corresponding to 0.31 ≤ $A_{750}^{7}$ ≤ 2.54. Overall we recognized five major outcomes: (1) negligible growth by both EHB+ and EHB− strains ($A_{750}^{7}$ ≤ 0.3), (2) measurable growth by both, but no difference in growth between EHB+ and EHB− strains, (3) measurable growth by the EHB+ strain but negligible growth by the EHB− strain, (4) measurable growth by both, with the EHB− strain reaching a higher density, and (5) measurable growth by both, with the EHB+ strain reaching a higher density.

### Statistical analyses

For preliminary assays, we compared colony diameter and spore production between EHB+ and EHB− strains using Welch *t*-tests. For Biolog® assays, we compared differences in global substrate use (i.e., growth across all substrates) between EHB+ and EHB− strains of *F. keratoplasticum* PS0362A using hierarchical clustering and permutational multivariate analysis of variance (PERMANOVA; Anderson, 2001; Anderson and ter Braak, 2003) based on the Bray-Curtis dissimilarity metric, as implemented in the R package *vegan* (R Core Team, 2015; Oksanen et al., 2016). For each time point, we first calculated dissimilarities among all replicate plates considering growth across all substrates. We then visualized global differences in substrate use among plates by constructing cluster dendrograms, and analyzed differences between plates inoculated with EHB+ and EHB− strains using PERMANOVA (*n* = 1,000 permutations). We further explored differences using non-parametric analysis of similarity (ANOSIM; *n* = 1,000 permutations; Clarke and Green, 1988; Clarke, 1993) and multi-response permutation procedures (MRPP; *n* = 1,000 permutations; Biondini et al., 1985), but given the normality of the data we focus here on results from PERMANOVA. We made comparisons for each time point in order to determine the time at which the global effect was largest. We then used that time point to evaluate differences in *A*_750_ between EHB+ vs. EHB− strains for focal substrates using Welch *t*-tests. We controlled for the rate of type I errors inherent in making multiple comparisons by using the false discovery rate-controlling method of Benjamini and Hochberg (1995). The raw data and R scripts for all analyses are available online (Shaffer, 2017).

## Results

Naturally infected (EHB+) and cured (EHB−) strains of *F. keratoplasticum* PS0362A were stable under laboratory conditions and grew readily on standard growth media and on diverse substrates in Biolog® assays. Both colony diameter and spore production by EHB+ and EHB− strains were similar on 2% MEA (Supplementary Figures 4, 5). However, in Biolog® assays, growth by the fungus was significantly influenced by the presence of the EHB *Chitinophaga* sp. PS-EHB01 across the majority of carbon sources (Figure 3 and Supplementary Figure 6, Table 1).

**Table 1:** **Comparison of total absorbance at 750 nm at 7 d ($A_{750}^{7}$) between EHB+ and EHB− strains of ***F. keratoplasticum*** PS0362A**.

	**EHB+**		**EHB−**		**Welch *t*-test**			
**Substrate**	**Mean**	***SD***	**Mean**	***SD***	***t***	***DF***	***p*-value (adj.)**	**Outcome**
**MONOSACCHARIDES**								
α-D-glucose	**1.49**	0.02	1.16	0.04	14.63	6.21	**<0.00001**	5
D-arabinose	0.71	0.04	**1.04**	0.07	9.31	6.90	**<0.00001**	4
D-fructose	1.69	0.18	1.47	0.14	2.20	7.60	0.08	2
D-galactose	**1.80**	0.08	1.41	0.10	6.75	7.38	**0.0004**	5
D-mannose	**1.58**	0.05	1.18	0.07	10.90	7.09	**<0.00001**	5
D-psicose	0.25	0.01	0.15	0.00	19.99	4.90	NA	1
D-ribose	**1.91**	0.06	1.23	0.18	8.00	4.84	**0.001**	5
D-tagatose	0.15	0.01	0.13	0.01	2.73	7.29	NA	1
D-xylose	**2.06**	0.06	1.39	0.05	19.12	7.44	**<0.000001**	5
L-arabinose	**1.72**	0.08	1.53	0.07	3.86	7.67	**0.008**	5
L-fucose	1.81	0.26	1.51	0.06	2.55	4.40	0.08	2
L-sorbose	**2.14**	0.03	1.44	0.05	30.13	6.44	**<0.0000001**	5
**DISACCHARIDES**								
α-D-lactose	**0.91**	0.08	0.39	0.02	14.44	4.76	**0.0001**	5
D-cellobiose	**1.61**	0.18	1.19	0.04	5.08	4.32	**0.009**	5
D-melibiose	**1.55**	0.04	1.38	0.04	6.00	7.99	**0.0007**	5
D-trehalose	**2.55**	0.01	2.15	0.04	21.83	5.05	**<0.00001**	5
gentiobiose	**1.71**	0.05	0.80	0.03	35.20	7.28	**<0.0000001**	5
isomaltulose	**1.69**	0.04	1.19	0.04	19.49	7.92	**<0.0000001**	5
lactulose	**0.66**	0.04	0.12	0.04	20.91	7.98	**<0.0000001**	3
maltose	**1.59**	0.03	1.38	0.03	10.51	7.84	**<0.00001**	5
sucrose	**1.92**	0.06	1.30	0.09	13.28	6.90	**<0.00001**	5
turanose	**2.13**	0.04	1.62	0.05	17.54	7.83	**<0.000001**	5
**TRISACCHARIDES**								
D-melezitose	**1.81**	0.06	1.34	0.04	14.53	7.38	**<0.000001**	5
D-raffinose	**1.46**	0.04	1.34	0.06	3.93	7.03	**0.009**	5
maltotriose	**1.54**	0.04	1.39	0.05	4.76	7.58	**0.003**	5
**TETRASACCHARIDES**								
stachyose	**1.52**	0.05	1.35	0.11	3.03	5.76	**0.03**	5
**OLIGOSACCHARIDES**								
dextrin	**0.91**	0.03	0.76	0.03	7.45	7.72	**0.0002**	5
**CYCLIC OLIGOSACCHARIDES**								
α-cyclodextrin	0.14	0.01	0.11	0.00	9.26	7.35	NA	1
β-cyclodextrin	0.38	0.08	0.32	0.04	1.74	6.39	0.2	2
**NUCLEOSIDES**								
adenosine	0.77	0.59	0.64	0.40	0.41	6.97	0.7	2
uridine	0.14	0.01	0.13	0.01	1.52	5.67	NA	1
**POLYSACCHARIDES**								
glycogen	**0.96**	0.03	0.81	0.02	8.57	6.03	**0.0003**	5
**SUGAR ALCOHOLS**								
D-arabitol	**2.28**	0.07	1.64	0.09	12.81	7.71	**<0.00001**	5
D-mannitol	2.44	0.06	2.36	0.14	1.29	5.54	0.3	2
D-sorbitol	**2.54**	0.03	1.45	0.36	6.67	4.05	**0.004**	5
glycerol	**1.68**	0.07	1.37	0.05	7.84	6.81	**0.0003**	5
*i*-erythritol	1.95	0.44	1.92	0.14	0.15	4.77	0.9	2
*myo*-inositol	**1.45**	0.04	1.18	0.02	12.56	6.01	**<0.00001**	5
maltitol	**2.12**	0.04	1.32	0.17	10.08	4.40	**0.0007**	5
ribitol	**1.18**	0.06	0.32	0.02	31.43	5.13	**<0.000001**	5
xylitol	0.17	0.01	0.17	0.01	0.88	6.07	NA	1
**METHYL SUGARS**								
L-rhamnose	**2.05**	0.04	1.79	0.07	7.72	6.17	**0.0005**	5
methyl α-D-galactoside	**1.89**	0.29	1.32	0.16	3.90	6.18	**0.01**	5
methyl β-D-galactoside	**0.58**	0.04	0.34	0.02	13.42	5.73	**<0.00001**	5
methyl α-D-glucoside	2.14	0.04	2.20	0.07	1.77	6.71	0.1	2
methyl β-D-glucoside	**2.21**	0.06	1.52	0.06	18.02	7.97	**<0.000001**	5
**ALCOHOLIC** β**-GLUCOSIDES**								
salicin	**0.97**	0.02	0.54	0.02	30.08	8.00	**<0.0000001**	5
**GLYCOSIDES**								
arbutin	**0.90**	0.06	0.70	0.03	7.16	5.99	**0.0007**	5
**MISC. CARBOHYDRATES**								
sedoheptulosan	0.12	0.01	0.14	0.03	1.63	4.77	NA	1
**AMINO SUGARS**								
D-glucosamine	0.39	0.11	**0.59**	0.05	3.69	5.71	**0.02**	4
*N*-acetyl-D-galactosamine	0.11	0.00	0.10	0.00	7.00	5.82	NA	1
*N*-acetyl-D-glucosamine	**2.38**	0.08	1.06	0.04	34.01	5.89	**<0.0000001**	5
*N*-acetyl-D-mannosamine	0.11	0.00	0.10	0.00	3.04	5.77	NA	1
**AMINO ACIDS**								
L-alanine	**1.65**	0.13	0.95	0.20	6.61	6.75	**0.0007**	5
L-proline	**2.12**	0.04	1.24	0.20	9.86	4.31	**0.0008**	5
L-serine	**1.50**	0.11	0.79	0.23	6.36	5.71	**0.002**	5
L-threonine	**1.97**	0.04	0.79	0.01	65.09	4.49	**<0.000001**	5
L-aspartic acid	1.18	0.06	1.18	0.11	0.03	5.92	1.0	2
L-glutamic acid	**1.51**	0.06	0.88	0.04	18.40	6.94	**<0.000001**	5
*N*-acetyl-L-glutamic acid	0.13	0.00	0.13	0.01	0.34	6.90	NA	1
glycyl-L-glutamic acid	0.13	0.00	0.13	0.01	1.79	7.22	NA	1
L-pyroglutamic acid	**1.77**	0.07	1.25	0.12	8.26	6.39	**0.0003**	5
γ-aminobutyric acid	**2.24**	0.03	0.84	0.12	24.28	4.63	**<0.00001**	5
L-asparagine	**1.86**	0.02	0.91	0.11	18.56	4.35	**<0.00001**	5
L-phenylalanine	1.21	0.05	1.23	0.29	0.12	4.26	0.9	2
L-ornithine	**1.81**	0.03	0.92	0.15	13.18	4.24	**0.0003**	5
L-alanyl-glycine	1.09	0.05	1.06	0.07	0.61	7.64	0.6	2
**AMINO ACID DERIVATIVES**								
amygdalin	**1.03**	0.04	0.83	0.03	8.55	7.63	**0.0001**	5
**CARBOXYLIC ACIDS**								
2-keto-D-gluconic acid	**0.84**	0.02	0.70	0.07	4.43	4.46	**0.01**	5
α-ketoglutaric acid	0.69	0.06	0.62	0.13	1.16	5.38	0.3	2
β-hydroxybutyric acid	**1.09**	0.07	0.72	0.06	9.35	7.90	**<0.00001**	5
D-glucuronic acid	**0.79**	0.04	0.49	0.06	9.03	7.21	**0.0001**	5
D-galacturonic acid	**1.14**	0.07	0.92	0.07	5.10	8.00	**0.002**	5
D-gluconic acid	**0.98**	0.02	0.67	0.06	11.77	4.84	**0.0002**	5
D-saccharic acid	**0.87**	0.04	0.57	0.04	13.52	7.99	**<0.000001**	5
D-malic acid	1.23	0.04	**1.47**	0.04	9.28	7.91	**<0.00001**	4
L-malic acid	0.81	0.02	**0.94**	0.09	3.06	4.40	**0.04**	4
L-lactic acid	**0.54**	0.02	0.40	0.02	11.45	7.91	**<0.00001**	5
γ-hydroxybutyric acid	0.23	0.10	0.15	0.01	1.71	4.08	NA	1
fumaric acid	1.09	0.05	1.13	0.15	0.62	4.99	0.6	2
p-hydroxyphenylacetic acid	1.46	0.08	1.24	0.24	1.94	4.92	0.1	2
quinic acid	**1.89**	0.09	1.15	0.17	8.41	6.04	**0.0003**	5
sebacic acid	**1.43**	0.06	0.94	0.15	6.70	5.05	**0.002**	5
succinic acid	**0.70**	0.02	0.52	0.05	7.70	4.90	**0.001**	5
**ESTERS**								
D-lactic acid methyl ester	0.22	0.00	0.17	0.00	15.96	8.00	NA	1
succinic acid monomethyl ester	**1.20**	0.11	0.79	0.16	4.76	6.87	**0.004**	5
**AMIDES**								
alaninamide	0.21	0.01	0.20	0.01	1.14	5.81	NA	1
glucuronamide	0.10	0.01	0.11	0.00	3.21	5.48	NA	1
succinamic acid	1.13	0.10	**1.82**	0.13	9.78	7.48	**<0.00001**	4
ethanolamine	0.92	0.04	1.05	0.21	1.41	4.23	0.3	1
putrescine	**1.90**	0.06	0.77	0.18	13.66	4.76	**0.0001**	5
**PHOSPHORYLATED CHEMICALS**								
glucose-1-phosphate	0.14	0.01	0.14	0.00	0.35	7.71	NA	1
adenosine-5′-monophosphate	0.12	0.01	0.11	0.00	2.28	5.97	NA	1
**BROMINATED CHEMICALS**								
bromosuccinic acid	**0.38**	0.02	0.25	0.01	13.54	7.90	**<0.000001**	3
**SURFACTANTS**								
Tween® 80	0.62	0.06	0.55	0.03	2.25	5.63	0.09	2
water	0.11	0.00	0.10	0.00	2.98	6.86	NA	1

*Values are means and standard deviations. Underlined means indicate substrates for which absorbance was negligible for both strains (i.e., $A_{750}^{7}$ ≤ 0.3). For the remaining substrates, results of Welch t-tests are shown. Adjusted p-values were corrected following Benjamini and Hochberg (1995). Significant p-values (i.e., ≤0.05) are bolded. Means in bold are those that were found to be significantly greater in comparisons. For each substrate, one of five observed outcomes is listed (see Section Results)*.

Overall the EHB+ strain used 79 of 95 carbon sources (Figure 3, Table 1). The EHB− strain used 77 of 95 of carbon sources, including all of those used by the EHB+ strain except for one disaccharide and one brominated chemical (see below; Figure 3 and Supplementary Figure 6, Table 1).

**Figure 4:**
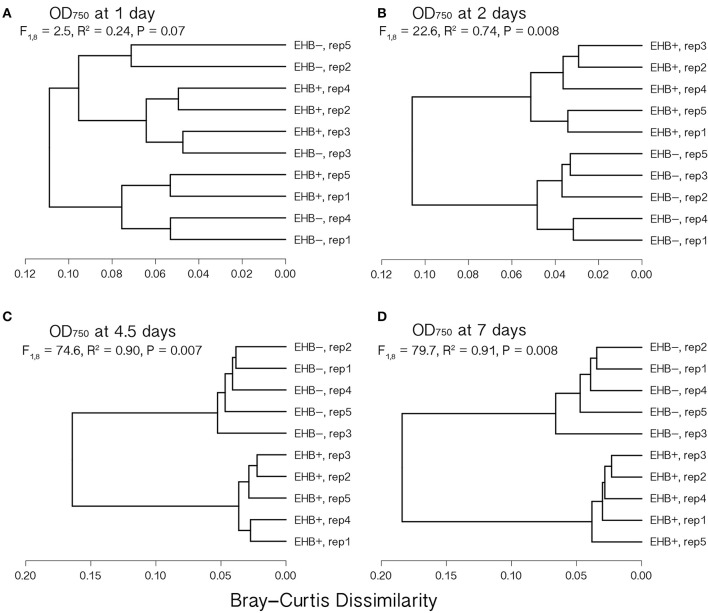
**Cluster dendrograms summarizing differences in global substrate use at 1, 2, 4.5, and 7 d among EHB+ and EHB− replicate Biolog® PMs**. Distances represent Bray-Curtis dissimilarities. Results from a permutational multivariate analysis of variance (PERMANOVA) are shown for each time point. Panels show differences at **(A)** 1 d, **(B)** 2 d, **(C)** 4.5 d, and **(D)** 7 d.

Global substrate use (i.e., growth across all substrates considered simultaneously) differed significantly between EHB+ and EHB− strains after 1 day and differentiated further throughout the remainder of the experiment (Figure 4, Table 2). For many substrates the initial growth rates of EHB+ and EHB− strains were similar; however, the hyphal densities at which EHB− strains reached stationary phase were often significantly lower than those at which EHB+ strains reached stationary phase (e.g., *N*-acetyl-D-glucosamine, L-proline, γ-aminobutyric acid, L-ornithine, quinic acid, D-gluconic acid, putrescine; Figure 3, Table 1). PERMANOVA indicated that global differences were greatest at 7 d (Figure 4D, Table 2); therefore, we used this time point for comparing growth between EHB+ and EHB− strains on individual substrates. We classified our results into four general outcomes, as described below.

**Table 2:** **Summary of differences in global substrate use between EHB+ and EHB− strains over 7 d**.

	**PERMANOVA**			**ANOSIM**		**MRPP**	
**Day**	***F***	***R*^2^**	***p*-value**	***R***	***p*-value**	***A***	***p*-value**
0.5	0.97	0.11	0.4	0.004	0.5	<0.0	0.5
1.0	2.48	0.24	0.07	0.33	0.07	0.07	0.08
1.5	6.86	0.46	**0.009**	1.00	**0.003**	0.23	**0.02**
2.0	22.60	0.74	**0.008**	1.00	**0.005**	0.42	**0.01**
2.5	18.10	0.69	**0.009**	1.00	**0.01**	0.40	**0.009**
3.0	35.19	0.81	**0.008**	1.00	**0.01**	0.51	**0.009**
3.5	57.42	0.88	**0.01**	1.00	**0.007**	0.59	**0.005**
4.0	75.49	0.90	**0.007**	1.00	**0.01**	0.63	**0.01**
4.5	74.62	0.90	**0.007**	1.00	**0.008**	0.63	**0.01**
5.0	75.12	0.90	**0.01**	1.00	**0.01**	0.64	**0.006**
5.5	71.26	0.90	**0.01**	1.00	**0.004**	0.63	**0.007**
6.0	72.46	0.90	**0.01**	1.00	**0.009**	0.63	**0.02**
6.5	78.24	0.91	**0.006**	1.00	**0.007**	0.64	**0.01**
7.0	79.72	0.91	**0.008**	1.00	**0.01**	0.65	**0.01**

*Bray-Curtis dissimilarities based on growth across all substrates were calculated for each pair of replicate phenotypic microarrays, which were grouped based on inoculation with EHB+ vs. EHB− strains. Results from three different methods (permutational multivariate analysis of variance [PERMANOVA], analysis of similarity [ANOSIM], and multi-response permutation procedure [MRPP]) confirm that significant differences in global substrate use between EHB+ and EHB– strains were observed and maintained after one day. Significant p-values (i.e., ≤0.05) are bolded*.

The 17 substrates on which we observed negligible growth by both the EHB+ and EHB− strains (Outcome 1) included diverse monosaccharides (e.g., D-psicose), amino sugars (e.g., *N*-acetyl-D-galactosamine), amino acids (e.g., glycyl-L-glutamic acid), amides (e.g., alaninamide), and phosphorylated chemicals (e.g., glucose-1-phosphate), in addition to water (Figure 3, Table 1).

EHB+ and EHB− strains both used, but did not differ in growth on, 15 substrates (Outcome 2; Figure 3, Table 1). These included diverse monosaccharides (e.g., D-fructose), sugar alcohols (e.g., D-mannitol), amino acids (e.g., phenylalanine), and carboxylic acids (e.g., fumaric acid).

On two substrates we observed measurable growth by the EHB+ strain and negligible growth by the EHB− strain (Outcome 3). These were lactulose and bromosuccinic acid, on which the EHB+ strain only grew to $A_{750}^{7}$ = 0.66 and 0.39, respectively (Figure 3, Table 1).

We observed measurable and significantly different growth between EHB+ and EHB− strains on 64 carbon sources (Figure 3, Table 1). The EHB− strain grew to a higher density on five substrates (Outcome 4), including one monosaccharide (D-arabinose), two stereoisomeric forms of one carboxylic acid (D- and L-malic acid), and one amide (succinamic acid; Figure 3, Table 1). The EHB+ strain grew to a higher density on 59 substrates (Outcome 5), including over three-quarters of all sugar-based substrates (77%), and most amino- and carboxylic acids and their derivatives (58 and 60%, respectively) (Figure 3, Table 1).

We repeated the experiment by re-curing the naturally infected strain and performing the Biolog® trial a second time. The raw data and code for analyses are available online (Shaffer, 2017). Results were consistent with those reported here.

## Discussion

Endohyphal bacteria (EHB) have been documented as symbionts in phylogenetically and ecologically diverse lineages of fungi (Barbieri et al., 2000; Bianciotto et al., 2003; Bertaux et al., 2005; Partida-Martínez et al., 2007b; Sharma et al., 2008; Hoffman and Arnold, 2010; Sato et al., 2010; Desirò et al., 2015; Shaffer et al., 2016). Only in a few cases have their effects been explored. Comparative genomics and phenotypic assays have recently highlighted the importance of certain proteobacterial EHB among foliar endophytic Ascomycota (Arendt, 2015; Baltrus et al., 2016). Here we provide the first insight to the influence of EHB on broad-spectrum substrate use by a member of a clade of fungi known for their widespread pathogenicity on plants (i.e., the *F. solani* species complex), with a focus on a strain affiliated with seeds from tropical forest soil. The strain considered here is a member of a lineage that is known for ecologically and medically important strains (i.e., *F. keratoplasticum*; Short et al., 2013). Its endohyphal bacterium belongs to a genus known for their production of secondary metabolites with antimicrobial activity (i.e., *Chitinophaga*). More broadly, it is a member of the Bacteroidetes, a phylogenetically diverse phylum of Gram-negative bacteria that are globally distributed, exhibit many biological functions, and are well-known symbionts of mammals and insects as well as degraders of organic matter (Moran et al., 2005; Thomas et al., 2011). Similar to Proteobacteria, Bacteroidetes are often one of the most representative taxa recovered from environmental sampling of freshwater, soil, animals guts and skin, and especially the phyllosphere (Redford et al., 2010; Thomas et al., 2011). To our knowledge, no endohyphal member of the Bacteroidetes has been examined previously for its associations with fungi or its phenotypic effects on a fungal host.

### Phenotypic microarrays

Phenotypic microarrays (PMs) provide a means to obtain quantitative data in a reproducible and highly controlled manner with respect to biomass accumulation from the metabolism of specific compounds. Such data can inform diverse and emerging fields concerning microbial ecology such as community systems biology and metametabolomics, and can provide the basis for hypotheses that can be evaluated in the context of genomics and transcriptomics analyses.

Recently PMs have been used to address questions in fungal ecology and evolution, including those relevant to biotechnology applications (Greetham, 2014; Blumenstein et al., 2015a), evolutionary relationships and species concepts (Rice and Currah, 2005; Atanasova et al., 2010), carbon dynamics and niche differentiation (Lee and Magan, 1999; Hobbie et al., 2003; Friedl et al., 2008), genetic and functional diversity (Dobranic and Zak, 1999; Druzhinina et al., 2006; Grizzle and Zak, 2006; Friedl et al., 2008), and ecophysiology (Druzhinina et al., 2010). Here we used a Biolog® plate assay as a rapid and simple method for use in characterizing the influence of EHB on broad-spectrum carbon source use by a filamentous fungus.

We found that the presence of *Chitinophaga* sp. PS-EHB01 significantly influenced substrate use across over two-thirds (67%) of carbon sources. The EHB+ strain grew to a higher absorbance compared to the EHB− strain across the majority of substrates (62%). In general, initial growth rates of EHB+ and EHB− strains were similar; however, the absorbance values at times after which the EHB− strain reached stationary phase were significantly lower than for those for when the EHB+ strain reached stationary phase (Figure 3, Table 1). We speculate that the bacterium may serve as a metabolic enhancer, possibly releasing compounds that serve as growth factors for the host fungus, or by detoxifying or metabolizing otherwise harmful waste or growth byproducts such as reactive oxygen species known to accumulate from catabolism of certain compounds (e.g., L-ornithine, putrescine; Pegg and Casero, 2011; Salvioli et al., 2016; Vannini et al., 2016). That *Chitinophaga* sp. PS-EHB01 appears to be consistently associated with *F. keratoplasticum* PS0362A but cannot be isolated into pure culture on standard media (Shaffer, unpublished) suggests that this EHB may rely on its host fungus to acquire essential nutrients.

### Perspectives from related species

Although their genomes have not yet been sequenced, genomic data are available for close relatives of the bacterium and fungus evaluated here (see Coleman et al., 2008; Del Rio et al., 2010). The focal bacterium is closely related to *Chitinophaga pinensis* (Supplementary Figure 7), which was isolated originally from soil and is known for its ability to degrade chitin (Sangkhobol and Skerman, 1981). *Chitinophaga pinensis* also produces antibiotics with activity against a diversity of filamentous fungi (Mohr et al., 2015). That species possesses multiple genes predicted to be involved in the metabolism of carbohydrates (*n* = 330) and amino acids (*n* = 301; Del Rio et al., 2010). The focal bacterium is also closely related to *C. arvensicola* (Supplementary Figure 7), a bacterium associated with amphibian skin that produces metabolites with inhibitory effects on the notorious fungal pathogen *Batrachochytrium dendrobatidis*, causal agent of chytridiomycosis (Loudon et al., 2014). Whether such traits are common in the *Chitinophaga* strain examined here remains to be determined.

Using a three-locus dataset, we showed previously that *F. keratoplasticum* PS0362A is part of the *F. solani* species complex (FSSC). The fungus studied here is closely related to FSSC Clade 3 haplotype group 2 (Shaffer et al., 2016). The closest relative with publicly available genomic data, *Nectria haematococca* MPVI, is in group 11-c (Short et al., 2013; Shaffer et al., 2016). *Nectria haematococca* MPVI occurs as a saprotroph and plant pathogen in diverse habitats (Coleman et al., 2008). Many members of the FSSC have conditionally dispensable, supernumerary chromosomes (CD chromosomes) that can influence the use of specific carbon sources (Covert, 1998; Coleman et al., 2008). CD chromosomes are mitotically stable in *N. haematococca* MPVI (Covert, 1998). Therefore, it is likely that the phenotypic results observed here do not reflect differential presence of CD chromosomes between EHB+ and EHB− strains, but rather a difference in the presence of the bacterium between them.

The genome of *N. haematococca* is highly enriched with genes coding for carbohydrate-active enzymes, including glycoside hydrolase and polysaccharide lyase genes (Coleman et al., 2008). *Nectria haematococca* also possesses a high number of ATP-binding cassette (ABC) transporter genes, second only to *Aspergillus oryzae* when compared to 10 other members of the Dikarya (Coleman et al., 2008). That the fungus studied here was able to use the majority of carbon sources regardless of EHB infection status may reflect similar gene composition. These substrates included some synthetic, non-natural compounds such as lactulose, bromosuccinic acid, and Tween® 80, emphasizing the metabolic breadth of this fungus. Strikingly, that breadth is increased markedly by the presence of *Chitinophaga* sp. PS-EHB01 as an EHB. Once data for both the bacterium and fungus are available, comparative genomics and transcriptomics can be used to understand metabolic interactions between the pair. More broadly, the pair could be developed to become a model system for understanding EHB of plant-associated Ascomycota.

### Implications for seed-fungus interactions

Fungi recruit from soil to seeds that have been dispersed to the soil seed bank, thus undergoing horizontal transmission (rather than being vertically transmitted from mother to offspring) (U'Ren et al., 2009; Sarmiento et al., 2015; Zalamea et al., 2015; Sarmiento et al., unpublished). Given this life history, seed-fungus interactions at the soil-seed interface (i.e., those involving the seed coat) are of primary interest with regard to community assembly of fungi in seeds. Furthermore, similar to bud-break or wounding (Agrios, 1997; Schädel et al., 2009; Gordon and Leveau, 2010; Savatin et al., 2014), seed germination represents a key event during which nutrients that may attract potential symbionts are released into the environment. The potential for EHB to influence seed-fungus interactions during colonization of seeds by fungi at the soil-seed interface, and during key plant life-stage transitions such as seed germination, should be investigated further.

Here we showed that the presence vs. absence of *Chitinophaga* sp. PS-EHB01 led to differential growth by *F. keratoplasticum* on most sugars, amino acids, and carboxylic acids, nearly all of which are relevant in plant biology. In particular, a number of substrates are important in the ecology of seeds, such as important global regulators (e.g., D-trehalose and *myo*-inositol; Loewus and Murthy, 2000; Grennan, 2007; Henry et al., 2014; Lunn et al., 2014), those metabolized or produced during seed imbibition and germination (e.g., D-trehalose, sucrose, D-raffinose, stachyose, dextrin, and L-asparagine; Atkins et al., 1975; Bewley and Black, 1978; Kuo et al., 1990; Queiroz and Cazetta, 2016), as well as those important in the metabolism of seed structural components such as the seed coat (e.g., D-mannose, L-arabinose, sucrose, D-raffinose, stachyose, *myo*-inositol, and L-alanine; Herold and Lewis, 1977; Bewley and Black, 1978; Kuo et al., 1990; Buckeridge et al., 2000; Loewus and Murthy, 2000; Lahuta et al., 2007; Kosina et al., 2013). The average difference in growth between EHB+ and EHB− strains, considering only those substrates on which we observed significant differences (*n* = 64; Table 1) was $A_{750}^{7}$ = 0.3. Whether this difference scales to meaningful changes with regard to interacting with plants in nature is not yet known, and will be assessed in future work using seed-infection and seed-germination experiments. We anticipate that changes in fungal substrate use by EHB will alter phenotypes that in turn define both the fungal niche and the outcomes of interactions with hosts.

## Author contributions

JU modified and tested the phenotypic microarray experimental protocol for use with fungal hyphal fragments; JS made additional modifications to exclude fungal spores, and performed all experimental work and related data analysis; AA and DB advised aspects of the data analysis; JS and AA led the development of the manuscript, with contributions from DB, RG, and JU.

## Funding

We thank the National Science Foundation (NSF DEB-1119758 to AA, NSF DEB-1120205 to James W. Dalling, NSF IOS-1354219 to DB, AA, and RG, NSF-IGERT Fellowship to JS), the Smithsonian Tropical Research Institute (STRI) (Short-term Fellowship to JS), the Mycological Society of America (Forest Fungal Ecology Award to JS), and the Graduate and Professional Student Council (Research Award to JS) and School of Plant Sciences (Pierson Fellowship to JS) at The University of Arizona for supporting this work. Additional support from the School of Plant Sciences and College of Agriculture and Life Sciences at the University of Arizona is gratefully acknowledged.

### Conflict of interest statement

The authors declare that the research was conducted in the absence of any commercial or financial relationships that could be construed as a potential conflict of interest.

## References

[B1] Agrios G. N. (1997). Plant Pathology. San Diego, CA: Academic Press.

[B2] Anca I. Lumini E. Ghignone S. Salvioli A. Bianciotto V. Bonfante P. (2009). The *ftsZ* gene of the endocellular bacterium ‘*Candidatus* Glomeribacter gigasporarum’ is preferentially expressed during the symbiotic phases of its host mycorrhizal fungus. Mol. Plant-Microbe Interact. 22, 302–310. 10.1094/MPMI-22-3-0302 19245324

[B3] Anderson M. J. (2001). A new method for non-parametric multivariate analysis of variance. Austral Ecol. 26, 32–46. 10.1111/j.1442-9993.2001.01070.pp.x

[B4] Anderson M. J. ter Braak C. J. F. (2003). Permutational tests for multi-factorial analysis of variance. J. Stat. Comput. Simul. 73, 85–113. 10.1080/00949650215733

[B5] Arendt K. A. (2015). Symbiosis Establishment and Ecological Effects of Endohyphal Bacteria on Foliar Fungi. Master's thesis, University of Arizona, Tucson, AZ.

[B6] Arendt K. A. Hockett K. L. Araldi-Brondolo S. J. Baltrus D. A. Arnold A. E. (2016). Isolation of endohyphal bacteria from foliar Ascomycota and *in vitro* establishment of their symbiotic associations. Appl. Environ. Microbiol. 82, 2943–2949. 10.1128/AEM.00452-16 26969692 PMC4959084

[B7] Arnold A. E. Engelbrecht B. M. J. (2007). Fungal endophytes nearly double minimum leaf conductance in seedlings of a neotropical tree species. J. Trop. Ecol. 23, 369–372. 10.1017/S0266467407004038

[B8] Arnold A. E. Lutzoni F. (2007). Diversity and host range of foliar fungal endophytes: are tropical leaves biodiversity hotspots? Ecology 88, 541–549. 10.1890/05-1459 17503580

[B9] Arnold A. E. Mejia L. C. Kyllo D. Rojas E. I. Maynard Z. Robbins N.. (2003). Fungal endophytes limit pathogen damage in a tropical tree. Proc. Natl. Acad. Sci. U.S.A. 100, 15649–15654. 10.1073/pnas.2533483100 14671327 PMC307622

[B10] Atanasova L. Druzhinina I. S. (2010). Global nutrient profiling by Phenotypic MicroArrays: a tool complementing genomic and proteomic studies in conidial fungi. J. Zhejiang Univ. Sci. B 11, 151–168. 10.1631/jzus.B1000007 20205302 PMC2833400

[B11] Atanasova L. Jaklitsch W. M. Komon-Zelazowska M. Kubicek C. P. Druzhinina I. S. (2010). Clonal species of *Trichoderma parareesei* sp. nov. likely resembles the ancestor of the cellulase producer *Hypocrea jecorina/T. reesei*. Appl. Environ. Microbiol. 76, 7259–7267. 10.1128/AEM.01184-10 20817800 PMC2976259

[B12] Atkins C. A. Pate J. S. Sharkey P. J. (1975). Asparagine metabolism – key to the nitrogen nutrition of developing legume seeds. Plant Physiol. 56, 807–812. 10.1104/pp.56.6.807 16659399 PMC541929

[B13] Baltrus D. A. Dougherty K. Arendt K. R. Huntemann M. Clum A. Pillay M. (2016). Absence of genome reduction in diverse, facultative endohyphal bacteria. Microb. Genom. [Epub ahead of print]. 10.1099/mgen.0.000101 PMC5361626 28348879

[B14] Barbieri E. Potenza L. Rossi I. Sisti D. Giomaro G. Rossetti S.. (2000). Phylogenetic characterization and *in situ* detection of *Cytophaga-Flexibacter-Bacteroides* phylogroup bacterium in *Tuber borchii* Vittad. ectomycorrhizal mycelium. Appl. Environ. Microbiol. 66, 5035–5042. 10.1128/AEM.66.11.5035-5042.2000 11055961 PMC92417

[B15] Benjamini Y. Hochberg Y. (1995). Controlling the false discovery rate: a practical and powerful approach to multiple testing. J. R. Stat. Soc. B 57, 289–300.

[B16] Bertaux J. Schmid M. Hutzler P. Hartmann A. Garbaye J. Frey-Klett P. (2005). Occurrence and distribution of endobacterial in the plant-associated mycelium of the ectomycorrhizal fungus *Laccaria bicolor* S238N. Environ. Microbiol. 7, 1786–1795. 10.1111/j.1462-2920.2005.00867.x 16232293

[B17] Bewley J. D. Black M. (1978). Physiology and Biochemistry of Seeds in Relation to Germination, Vol. 1. Development, Germination, and Growth. Berlin: Springer-Verlag.

[B18] Bianciotto V. Bandi C. Minerdi D. Sironi M. Tichy H. V. Bonfante P. (1996). An obligately endosymbiotic mycorrhizal fungus itself harbors obligately intracellular bacteria. Appl. Environ. Microbiol. 62, 3005–3010. 8702293 10.1128/aem.62.8.3005-3010.1996 PMC168087

[B19] Bianciotto V. Genre A. Jargeat P. Lumini E. Bécard G. Bonfante P. (2004). Vertical transmission of endobacteria in the arbuscular mycorrhizal fungus *Gigaspora margarita* through generation of vegetative spores. Appl. Environ. Microbiol. 70, 3600–3608. 10.1128/AEM.70.6.3600-3608.2004 15184163 PMC427789

[B20] Bianciotto V. Lumini E. Bonfante P. Vandamme P. (2003). ‘*Candidatus* Glomeribacter gigasporarum’ gen. nov., sp. nov., an endosymbiont of arbuscular mycorrhizal fungi. Int. J. Syst. Evol. Microbiol. 53, 121–124. 10.1099/ijs.0.02382-0 12656162

[B21] Biondini M. E. Bonham C. D. Redente E. F. (1985). Secondary successional patterns in a sagebrush (*Artemisia tridentata*) community as they relate to soil disturbance and soil biological activity. Vegetatio 60, 25–36. 10.1007/BF00053909

[B22] Blanchette R. A. (1991). Delignification by wood-decay fungi. Annu. Rev. Phytopathol. 29, 381–398. 10.1146/annurev.py.29.090191.002121

[B23] Blumenstein K. Albrectsen B. R. Martín J. A. Hultberg M. Sieber T. N. Helander M. (2015a). Nutritional niche overlap potentiates the use of endophytes in biocontrol of a tree disease. Biocontrol 60, 655–667. 10.1007/s10526-015-9668-1

[B24] Blumenstein K. Macaya-Sanz D. Martín J. A. Albrectsen B. R. Witzell J. (2015b). Phenotype MicroArrays as a complementary tool to next generation sequencing for characterization of tree endophytes. Front. Microbiol. 6:1033. 10.3389/fmicb.2015.01033 26441951 PMC4585013

[B25] Bochner B. R. (1989). Sleuthing out bacterial identities. Nature 339, 157–158. 10.1038/339157a0 2654644

[B26] Bochner B. R. (2003). New technologies to assess genotype-phenotype relationships. Nat. Rev. Genet. 4, 309–314. 10.1038/nrg1046 12671661

[B27] Bochner B. R. (2008). Global phenotypic characterization of bacteria. FEMS Microbiol. Rev. 33, 191–205. 10.1111/j.1574-6976.2008.00149.x 19054113 PMC2704929

[B28] Bochner B. R. Savageau M. A. (1977). Generalized indicator plate for genetic, metabolic, and taxonomic studies with microorganisms. Appl. Environ. Microbiol. 33, 434–444. 322611 10.1128/aem.33.2.434-444.1977 PMC170700

[B29] Bochner B. R. Gadzinksi P. Panomitros E. (2001). Phenotype MicroArrays for high-throughput phenotypic testing and assay of gene function. Genome Res. 11, 1246–1255. 10.1101/gr.186501 11435407 PMC311101

[B30] Buckeridge M. S. dos Santos H. P. Tiné M. A. S. (2000). Mobilisation of storage cell wall polysaccharides in seeds. Plant Physiol. Biochem. 38, 141–156. 10.1016/S0981-9428(00)00162-5

[B31] Busby P. E. Peay K. G. Newcombe G. (2015). Common foliar fungi of *Populus trichocarpa* modify *Melampsora* rust disease severity. New Phytol. 209, 1681–1692. 10.1111/nph.13742 26565565

[B32] Clarke K. R. (1993). Non-parametric multivariate analyses of changes in community structure. Austral Ecol. 18, 117–143. 10.1111/j.1442-9993.1993.tb00438.x

[B33] Clarke K. R. Green R. H. (1988). Statistical design and analysis for a ‘biological effects’ study. Mar. Ecol. Prog. Ser. 46, 213–226. 10.3354/meps046213

[B34] Coleman J. J. Rounsley S. D. Rodriguez-Carres M. Kuo A. Wasmann C. C. Grimwood J.. (2008). The genome of *Nectria haematococca*: contribution of supernumerary chromosomes to gene expansion. PLoS Genet. 5:e1000618. 10.1371/journal.pgen.1000618 19714214 PMC2725324

[B35] Covert S. F. (1998). Supernumerary chromosomes in filamentous fungi. Curr. Genet. 33, 311–319. 10.1007/s002940050342 9618581

[B36] Davison J. Moora M. Öpik M. Adholeya A. Ainsaar L. Bâ A.. (2015). Global assessment of arbuscular mycorrhizal fungus diversity reveals very low endemism. Science 349, 970–973. 10.1126/science.aab1161 26315436

[B37] Del Rio T. G. Abt B. Spring S. Lapidus A. Nolan M. Tice H.. (2010). Complete genome sequence of *Chitionphaga pinensis* type strain (UQM 2034^T^). Stand. Genomic Sci. 2, 87–95. 10.4056/sigs.661199 21304681 PMC3035255

[B38] Desirò A. Faccio A. Kaech A. Bidartondo M. I. Bonfante P. (2015). *Endogone*, one of the oldest plant-associated fungi, host unique Mollicutes-related endobacteria. New Phytol. 205, 1464–1472. 10.1111/nph.13136 25345989

[B39] Dobranic J. K. Zak J. C. (1999). A microtiter plate procedure for evaluating fungal functional diversity. Mycologia 91, 756–765. 10.2307/3761529 16894979

[B40] Druzhinina I. S. Komon-Zelazowska M. Atanasova L. Seidl V. Kubicek C. P. (2010). Evolution and ecophysiology of the industrial producer *Hypocrea jecorina* (Anamorph *Trichoderma reesei*) and a new sympatric agamospecies related to it. PLoS ONE 5:e9191. 10.1371/journal.pone.0009191 20169200 PMC2820547

[B41] Druzhinina I. S. Schmoll M. Seiboth B. Kubicek C. P. (2006). Global carbon utilization profiles of wild-type, mutant, and transformant strains of *Hypocrea jecorina*. Appl. Environ. Microbiol. 72, 2126–2133. 10.1128/AEM.72.3.2126-2133.2006 16517662 PMC1393202

[B42] Estrada C. Degner E. C. Rojas E. I. Wcislo W. T. Van Bael S. A. (2015). The role of endophyte diversity in protecting plants from defoliation by leaf-cutting ants. Curr. Sci. 109, 55–61.

[B43] Ewing B. Green P. (1998). Base-calling of automated sequencer traces using *Phred*. II. Error probabilities. Genome Res. 8, 186–194. 10.1101/gr.8.3.186 9521922

[B44] Ewing B. Hillier L. Wendl M. C. Green P. (1998). Base-calling of automated sequencer traces using *Phred*. I. Accuracy assessment. Genome Res. 8, 175–185. 10.1101/gr.8.3.175 9521921

[B45] Frey-Klett P. Garbaye J. Tarkka M. (2007). The mycorrhiza helper bacteria revisited. New Phytol. 176, 22–36. 10.1111/j.1469-8137.2007.02191.x 17803639

[B46] Friedl M. A. Kubicek C. P. Druzhinina I. S. (2008). Carbon source dependence and photostimulation of conidiation in *Hypocrea atroviridis*. Appl. Environ. Microbiol. 74, 245–250. 10.1128/AEM.02068-07 17981948 PMC2223218

[B47] Gale G. R. Harrington R. L. Pate A. F. (1960). The preparation of mycelial suspensions of dermatophytes for metabolic studies. J. Invest. Dermatol. 34, 167–169. 10.1038/jid.1960.22 13826035

[B48] Gallery R. E. Dalling J. W. Arnold A. E. (2007). Diversity, host affinity, and distribution of seed-infecting fungi: a case study with *Cecropia*. Ecology 88, 582–588. 10.1890/05-1207 17503585

[B49] Gallery R. E. Moore D. J. P. Dalling J. W. (2010). Interspecific variation in susceptibility to fungal pathogens in seeds of 10 tree species in the neotropical genus *Cecropia*. J. Ecol. 98, 147–155. 10.1111/j.1365-2745.2009.01589.x

[B50] Ghignone S. Salvioli A. Anca I.-A. Lumini E. Ortu G. Petiti L.. (2012). The genome of the obligate endobacterium of an AM fungus reveals an interphylum network of nutritional interactions. ISME J. 6, 136–145. 10.1038/ismej.2011.110 21866182 PMC3246228

[B51] Gordon T. R. Leveau J. H. J. (2010). Plant pathology: a story about biology. Annu. Rev. Phytopathol. 48, 293–309. 10.1146/annurev-phyto-080508-081919 19400651

[B52] Greetham D. (2014). Phenotype microarray technology and its application in industrial biotechnology. Biotechnol. Lett. 36, 1153–1160. 10.1007/s10529-014-1481-x 24563312

[B53] Grennan A. K. (2007). The role of trehalose biosynthesis in plants. Plant Physiol. 144, 3–5. 10.1104/pp.104.900223 17494918 PMC1913774

[B54] Grimmer M. K. Foulkes M. J. Paveley N. D. (2012). Foliar pathogenesis and plant water relations: a review. J. Exp. Bot. 63, 4321–4331. 10.1093/jxb/ers143 22664583

[B55] Grizzle H. W. Zak J. C. (2006). A microtiter plate procedure for evaluating fungal functional diversity on nitrogen substrates. Mycologia 98, 353–363. 10.3852/mycologia.98.2.353 16894979

[B56] Heilmann-Clausen J. Boddy L. (2008). Distribution patterns of wood-decay basidiomycetes at the landscape to global scale, in Ecology of Saprotrophic Basidiomycetes (British Mycological Society Symposia Series Volume 28), eds Boddy L. Frankland J. C. van West P. (London: Academic Press), 3–372.

[B57] Henry C. Bledsoe S. W. Siekman A. Kollman A. Waters B. M. Feil R.. (2014). The trehalose pathway in maize: conservation and gene regulation in response to the diurnal cycle and extended darkness. J. Exp. Bot. 65, 5959–5973. 10.1093/jxb/eru335 25271261 PMC4203130

[B58] Herold A. Lewis D. H. (1977). Mannose and green plants: occurrence, physiology and metabolism, and use as a tool to study the role of orthophosphate. New Phytol. 79, 1–40. 10.1111/j.1469-8137.1977.tb02178.x

[B59] Hobbie E. A. Watrud L. S. Maggard S. Shiroyama T. Rygiewicz P. T. (2003). Carbohydrate use and assimilation by litter and soil fungi assessed by carbon isotopes and BIOLOG® assays. Soil Biol. Biochem. 35, 303–311. 10.1016/S0038-0717(02)00281-X

[B60] Hoffman M. T. Arnold A. E. (2010). Diverse bacteria inhabit living hyphae of phylogenetically diverse fungal endophytes. Appl. Environ. Microbiol. 76, 4063–4075. 10.1128/AEM.02928-09 20435775 PMC2893488

[B61] Hoffman M. T. Gunatilaka M. K. Wijeratne K. Gunatilaka L. Arnold A. E. (2013). Endohyphal bacterium enhances production of indole-3-acetic acid by a foliar fungal endophyte. PLoS ONE 8:e73132. 10.1371/journal.pone.0073132 24086270 PMC3782478

[B62] Izumi H. Anderson I. C. Alexander I. J. Killham K. Moore E. R. B. (2005). Endobacteria in some ectomycorrhiza of Scotspine (*Pinus sylvestris*). FEMS Microbiol. Ecol. 56, 34–43. 10.1111/j.1574-6941.2005.00048.x 16542403

[B63] Jones J. D. G. Dangl J. L. (2006). The plant immune system. Nature 444, 323–329. 10.1038/nature05286 17108957

[B64] Kivlin S. N. Hawkes C. V. Treseder K. K. (2011). Global diversity and distribution of arbuscular mycorrhizal fungi. Soil Biol. Biogeochem. 43, 2294–2303. 10.1016/j.soilbio.2011.07.012

[B65] Kosina S. M. Schnebly S. R. Obendorf R. L. (2013). Are Raffinose and Stachyose unloaded from soybean seed coats to developing embryos? *Open Plant Sci*. J. 7, 10–16. 10.2174/1874294701307010010

[B66] Krieg N. R. Staley J. T. Brown D. R. Hedlund B. P. Paster B. J. Ward N. L. (2010). Volume Four: The Bacteroidetes, Spirochaetes, Tenericutes (Mollicutes), Acidobacteria, Fibrobacteres, Fusobacteria, Dictyoglomi, Gemmatimonadetes, Lentisphaerae, Verrucomicrobia, Chlamydiae, and Planctomycetes, in Bergey's Manual of Systematic Bacteriology 2nd Edn., eds Goodfellow M. Kämpfer P. Chun J. De Vos P. Rainey F. Whitman W. B. (New York, NY: Springer), 25–467.

[B67] Kubicek C. P. Bissett J. Druzhinina I. Kullnig-Gradinger C. Szakacs G. (2003). Genetic and metabolic diversity of *Trichoderma*: a case study on South-East Asian isolates. Fungal Genet. Biol. 38, 310–319. 10.1016/S1087-1845(02)00583-2 12684020

[B68] Kuo T. M. Doehlert D. C. Crawford C. G. (1990). Sugar metabolism in germinating soybean seeds. Plant Physiol. 93, 1514–1520. 10.1104/pp.93.4.1514 16667649 PMC1062704

[B69] Lahuta L. B. Górecki R. J. Zalewski K. Hedley C. L. (2007). Sorbitol accumulation during natural and accelerated ageing of pea (*Pisum sativum* L.) seeds. Acta Physiol. Plant 29, 527–534. 10.1007/s11738-007-0063-0

[B70] Lane D. J. (1991). 16S/23S rRNA sequencing, in Nucleic Acid Techniques in Bacterial Systematics, eds Stackebrandt E. Goodfellow M. (Chichester: John Wiley and Sons), 115–175.

[B71] Lee H. B. Magan N. (1999). Environmental factors and nutritional utilization patterns affect niche overlap indices between *Aspergillus ochraceus* and other spoilage fungi. Lett. Appl. Microbiol. 28, 300–304. 10.1046/j.1365-2672.1999.00521.x 10212444

[B72] Leigh E. G. Jr. (1999). Tropical Forest Ecology: A View from Barro Colorado Island. New York, NY: Oxford University Press.

[B73] Loewus F. A. Murthy P. P. N. (2000). *myo*-Inositol metabolism in plants. Plant Sci. 150, 1–19. 10.1016/S0168-9452(99)00150-8

[B74] Loudon A. H. Holland J. A. Umile T. P. Burzynski E. A. Minbiole K. P. C. Harris R. N. (2014). Interactions between amphibians' symbiotic bacteria cause the production of emergent anti-fungal metabolites. Front. Microbiol. 5:441. 10.3389/fmicb.2014.00441 25191317 PMC4139739

[B75] Lumini E. Bianciotto V. Jargeat P. Novero M. Salvioli A. Faccio A.. (2007). Presymbiotic growth and sporal morphology are affected in the arbuscular mycorrhizal fungus *Gigaspora margarita* cured of its endobacteria. Cell. Microbiol. 9, 1716–1729. 10.1111/j.1462-5822.2007.00907.x 17331157

[B76] Lunn J. E. Delorge I. Figueroa C. M. Van Dijck P. Stitt M. (2014). Trehalose metabolism in plants. Plant, J. 79, 544–567. 10.1111/tpj.12509 24645920

[B77] Maddison D. R. Maddison W. P. (2005). ChromaSeq Module. Mesquite: A Modular System for Evolutionary Analysis. Version 1.06. Available online at: http://mesquiteproject.org/

[B78] Maddison W. P. Maddison D. R. (2009). Mesquite: A Modular System for Evolutionary Analysis. Version 2.6. Available online at: http://mesquiteproject.org/

[B79] Márquez L. M. Redman R. S. Rodriguez R. J. Roossink M. L. (2007). A virus in a fungus in a plant: three-way symbiosis required for thermal tolerance. Science 315, 513–515. 10.1126/science.1136237 17255511

[B80] Mohr K. I. Volz C. Jansen R. Wray V. Hoffman J. Bernecker S.. (2015). Pinensins: the first antifungal lantibiotics. Angew. Chem. Int. Ed. 54, 11254–11258. 10.1002/anie.201500927 26211520

[B81] Moran N. A. Tran P. Gerardo N. M. (2005). Symbiosis and insect diversification: an ancient symbiosis of sap-feeding insects from the bacterial phylum Bacteroidetes. Appl. Environ. Microbiol. 71, 8802–8810. 10.1128/AEM.71.12.8802-8810.2005 16332876 PMC1317441

[B82] Oksanen J. Blanchet F. G. Kindt R. Legendre P. Minchin P. R. O'Hara R. B. (2016). vegan: Community Ecology Package. R Package Version 2.3-3. Available online at: http://CRAN.R-project.org/package=vegan

[B83] Oliva J. Stenlid J. Martínez-Vilalta J. (2014). The effect of fungal pathogens on the water and carbon economy of trees: implications for drought-induced mortality. New Phytol. 203, 1028–1035. 10.1111/nph.12857 24824859

[B84] Orbach M. J. Chumley F. G. Valent B. (1996). Electrophoretic karyotypes of *Magnaporthe grisea* pathogens of diverse grasses. Mol. Plant Microbe Interact. 9, 261–271. 10.1094/MPMI-9-0261

[B85] Partida-Martínez L. P. Groth I. Schmitt I. Richter W. Roth M. Hertweck C. (2007b). *Burkholderia rhizoxinica* sp. nov. and *Burkholderia endofungorum* sp. nov., bacterial endosymbionts of the plant-pathogenic fungus *Rhizopus microsporus*. Int. J. Syst. Evol. Microbiol. 57, 2583–2590. 10.1099/ijs.0.64660-0 17978222

[B86] Partida-Martínez L. P. Hertweck C. (2005). Pathogenic fungus harbours endosymbiotic bacteria for toxin production. Nature 437, 884–888. 10.1038/nature03997 16208371

[B87] Partida-Martínez L. P. Monajembashi S. Greulich K.-O. Hertweck C. (2007a). Endosymbiont-dependent host reproduction maintains bacterial-fungal mutualism. Curr. Biol. 17, 773–777. 10.1016/j.cub.2007.03.039 17412585

[B88] Pegg A. E. Casero R. A. Jr. (2011). Current status of the polyamine research field. Methods Mol. Biol. 720, 3–35. 10.1007/978-1-61779-034-8_1 21318864 PMC3652263

[B89] Pfliegler W. P. Atanasova L. Karanyicz E. Sipiczki M. Bond U. Druzhinina I. S. (2014). Generation of new genotypic and phenotypic features in artificial and natural yeast hybrids. Food Technol. Biotechnol. 52, 46–57. 10.1007/s00253-016-7481-0

[B90] Queiroz R. J. B. Cazetta J. O. (2016). Proline and trehalose in maize seeds germinating under low osmotic potentials. Rev. Bras. Eng. Agríc. Ambient. 20, 22–28. 10.1590/1807-1929/agriambi.v20n1p22-28

[B91] R Core Team (2015). R: A Language and Environment for Statistical Computing. Vienna: R Foundation for Statistical Computing Available online at: http://www.R-project.org/

[B92] Redford A. J. Bowers R. M. Knight R. Linhard Y. Fierer N. (2010). The ecology of the phyllosphere: geographic and phylogenetic variability in the distribution of bacteria on tree leaves. Environ. Microbiol. 12, 2885–2893. 10.1111/j.1462-2920.2010.02258.x 20545741 PMC3156554

[B93] Rice A. V. Currah R. S. (2005). Profiles from Biolog FF plates and morphological characteristics support recognition of *Oidiodendron fimicola* sp. nov. Stud. Mycol. 53, 75–82. 10.3114/sim.53.1.75

[B94] Ruiz-Herrera J. León-Ramírez C. Vera-Nu-ez A. Sánchez-Arreguín A. Ruiz-Medrano R. Salgado-Lugo H.. (2015). A novel intracellular nitrogen-fixing symbiosis made by *Ustilago maydis* and *Bacillus* spp. New Phytol. 207, 769–777. 10.1111/nph.13359 25754368

[B95] Salvioli A. Chiapello M. Fontaine J. Hadj-Sahraoui A. L. Grandmougin-Ferjani A. Lanfranco L.. (2010). Endobacteria affect the metabolic profile of their host *Gigaspora margarita*, an arbuscular mycorrhizal fungus. Environ. Microbiol. 12, 2083–2095. 10.1111/j.1462-2920.2010.02246.x 21966904

[B96] Salvioli A. Ghignone S. Novero M. Navazio L. Venice F. Bagnaresi P.. (2016). Symbiosis with an endobacterium increases the fitness of a mycorrhizal fungus, raising its bioenergetic potential. ISME J. 10, 130–144. 10.1038/ismej.2015.91 26046255 PMC4681866

[B97] Sangkhobol V. Skerman V. B. D. (1981). *Chitinophaga*, a new genus of chitinolytic myxobacteria. Int. J. Syst. Bacteriol. 31, 285–293. 10.1099/00207713-31-3-285

[B98] Sarmiento C. Zalamea P.-C. Dalling J. W. Davis A. S. Arnold A. E. (2015). Seed-associated fungi: effects on seed survival and germination of tropical pioneer species, in Conference for the Association for Tropical Biology and Conservation, July 12–16 (Honolulu, HI).

[B99] Sato Y. Narisawa K. Tsuruta K. Umezu M. Nishizawa T. Tanaka K.. (2010). Detection of Betaproteobacteria inside the mycelium of the fungus *Mortierella elongata*. Microbes Environ. 25, 321–324. 10.1264/jsme2.ME10134 21576890

[B100] Savatin D. V. Gramegna G. Modesti V. Cervone F. (2014). Wounding in the plant tissue: the defense of a dangerous passage. Front. Plant Sci. 5:470. 10.3389/fpls.2014.00470 25278948 PMC4165286

[B101] Schädel C. Blöchl A. Richter A. Hoch G. (2009). Short-term dynamics of nonstructural carbohydrates and hemicelluloses in young branches of temperate forest trees during bud break. Tree Physiol. 29, 901–911. 10.1093/treephys/tpp034 19457884

[B102] Schafer M. Kotanen P. M. (2003). The influence of soil moisture on losses of buried seeds to fungi. Acta Oecol. 24, 255–263. 10.1016/j.actao.2003.09.001

[B103] Schneider C. A. Rasband W. S. Eliceiri K. W. (2012). NIH to ImageJ: 25 years of image analysis. Nat. Methods 9, 671–675. 10.1038/nmeth.2089 22930834 PMC5554542

[B104] Shaffer J. (2017). Justinshaffer/Endohyphal_Bacterium_Alters_Substrate_Use_by_Fusarium_Keratoplasticum: Second Release of Code for Analyzing Biolog FF Microplate Data [Data Set]. Zenodo 10.5281/zenodo.250931

[B105] Shaffer J. P. Sarmiento C. Zalamea P.-C. Gallery R. E. Davis A. S. Baltrus D. A. (2016). Diversity, specificity, and phylogenetic relationships of endohyphal bacteria in fungi that inhabit tropical seeds and leaves. Front. Ecol. Evol. 4:116 10.3389/fevo.2016.00116

[B106] Sharma M. Schmid M. Rothballer M. Hause G. Zucarro A. Imanl J.. (2008). Detection and identification of bacteria intimately associated with fungi in the order Sebacinales. Cell. Microbiol. 10, 2235–2246. 10.1111/j.1462-5822.2008.01202.x 18637023

[B107] Short D. P. G. O'Donnell K. Thrane U. Nielson K. F. Zhang N. Juba J. H.. (2013). Phylogenetic relationships among members of the *Fusarium solani* species complex in human infections and the descriptions of *F. keratoplasticum* sp. nov. and *F. petroliphilum* stat. nov. Fungal Genet. Biol. 53, 59–70. 10.1016/j.fgb.2013.01.004 23396261

[B108] Singh M. P. (2009). Application of Biolog FF MicroPlate for substrate utilization and metabolite profiling of closely related fungi. J. Microbiol. Methods 77, 102–108. 10.1016/j.mimet.2009.01.014 19318055

[B109] Spraker J. E. Sanchez L. M. Lowe T. M. Dorrestein P. C. Keller N. P. (2016). *Ralstonia solanacearum* lipopeptide induces chlamydospore development in fungi and facilitates bacterial entry into fungal tissues. ISME J. 10, 2317–2330. 10.1038/ismej.2016.32 26943626 PMC4989320

[B110] Tanzer M. M. Arst H. N. Skalchunes A. R. Coffin M. Darveaux B. A. Heiniger R. W.. (2003). Global nutritional profiling for mutant and chemical mode-of-action analysis in filamentous fungi. Funct. Integr. Genomics 3, 160–170. 10.1007/s10142-003-0089-3 12898394

[B111] Tedersoo L. Bahram M. Põlme S. Kõljalg U. Yorou N. S. Wijesundera R.. (2014). Global diversity and geography of soil fungi. Science 346:1256688. 10.1126/science.1256688 25430773

[B112] Thomas F. Hehemann J.-H. Rebuffet E. Czjzek M. Michel G. (2011). Environmental and gut Bacteroidetes: the food connection. Front. Microbiol. 2:93. 10.3389/fmicb.2011.00093 21747801 PMC3129010

[B113] U'Ren J. (2016). DNA Extraction from Fungal Mycelium using Extract-n-Amp. Available online at: www.protocols.io

[B114] U'Ren J. M. Dalling J. W. Gallery R. E. Maddison D. R. Davis E. C. Gibson C. M.. (2009). Diversity and evolutionary origins of fungi associated with seeds of a neotropical pioneer tree: a case study for analyzing fungal environmental samples. Mycol. Res. 113, 432–449. 10.1016/j.mycres.2008.11.015 19103288

[B115] U'Ren J. M. Miadlikowska J. Zimmerman N. B. Lutzoni F. Stajich J. E. Arnold A. E. (2016). Contributions of North American endophytes to the phylogeny, ecology, and taxonomy of Xylariaceae (Sordariomycetes, Ascomycota). Mol. Phylogenet. Evol. 98, 210–232. 10.1016/j.ympev.2016.02.010 26903035

[B116] van der Heijden M. G. A. Martin F. M. Selosse M.-A. Sanders I. R. (2015). Mycorrhizal ecology and evolution: the past, the present, and the future. New Phytol. 205, 1406–1423. 10.1111/nph.13288 25639293

[B117] Vannini C. Carpentieri A. Salvioli A. Novero M. Marsoni M. Testa L.. (2016). An interdomain network: the endobacterium of a mycorrhizal fungus promotes antioxidative responses in both fungal and plant hosts. New Phytol. 211, 265–275. 10.1111/nph.13895 26914272

[B118] Waller F. Achatz B. Baltruschat H. Fodor J. Becker K. Fischer M.. (2005). The endophytic fungus *Piriformospora indica* reprograms barley to salt-stress tolerance, disease resistance, and higher yield. Proc. Natl. Acad. Sci. U.S.A. 102, 13386–13391. 10.1073/pnas.0504423102 16174735 PMC1224632

[B119] Zalamea P.-C. Sarmiento C. Arnold A. E. Davis A. S. Dalling J. W. (2015). Do soil microbes and abrasion by soil particles influence persistence and loss of physical dormancy in seeds of tropical pioneers? Front. Plant Sci. 5:799. 10.3389/fpls.2014.00799 25628640 PMC4292399

