# Supplementary material for: An Endohyphal Bacterium (Chitinophaga, Bacteroidetes) Alters Carbon Source Use by Fusarium keratoplasticum (F. solani Species Complex, Nectriaceae)
Source: Front Microbiol. 2017 Mar 14;8:350. doi: 10.3389/fmicb.2017.00350 (PMC5361657; doi:10.3389/fmicb.2017.00350)
Supplement: Supplementary file 1 [file DataSheet1.pdf]

Supplementary Material

An Endohyphal Bacterium (*Chitinophaga*, Bacteroidetes) Alters Carbon Source Use by *Fusarium keratoplasticum* (*F. solani* species complex, Nectriaceae)

Justin P. Shaffer, Jana M. U'Ren, Rachel E. Gallery, David A. Baltrus, and A. Elizabeth Arnold*

*** Correspondence:** Dr. A. Elizabeth Arnold: Arnold@ag.arizona.edu

1 Supplementary Materials and Methods

The focal endohyphal bacterium investigated here was identified previously by phylogenetic analysis of the 16*S* rRNA gene as *Chitinophaga* sp. (Bacteroidetes) (Shaffer et al., 2016). However the only other described congener included in that analysis was its closest BLAST match, *C. sancti.* We analyzed additional species of *Chitinophaga* here to refine the placement of the EHB within the Chitinophagaceae and specifically with respect to described *Chitinophaga* spp. We followed the taxon sampling of Proença et al. (2014), but chose two members of the Saprospiraceae – the only other family in the Chitinophagales, rather than those belonging to the more distantly related Flavobacteriales – as the outgroup. Overall, our dataset included 26 reference sequences from GenBank in addition to the sequence from the focal endohyphal *Chitinophaga*.

Methods for phylogenetic analyses followed Shaffer et al. (2016). Briefly, we first used Muscle v3.7 (Edgar, 2004) to align sequences and then edited the alignment by eye in Mesquite v2.75 (Maddison and Maddison, 2009). Ambiguously aligned characters were excluded, resulting in a final alignment of 1,342 characters. Maximum likelihood analysis was conducted in RAxML v8.2.4 (Stamatakis, 2014) with bootstrap support determined from 1,000 replicates. Bayesian analysis was conducted in MrBayes v3.2.6 (Huelsenbeck and Ronquist, 2001; Ronquist and Huelsenbeck, 2003) with 20 million generations, initiated with random trees, four chains, sampling every 1,000th tree, and a burn-in of all trees with a standard deviation of split frequencies ≥ 0.01. For both analyses, we used jModelTest2 (Posada, 2008; Darriba et al., 2012) to select the appropriate model of sequence evolution (i.e., GTR+Γ+I). Muscle, RAxML, MrBayes, and jModelTest2 were implemented using the CIPRES Science Gateway (Miller et al., 2010).

2 Supplementary Data

We have published the raw data and R script for all analyses online (Shaffer, 2017).

3 Supplementary Figures and Tables

3.1 Supplementary Figures

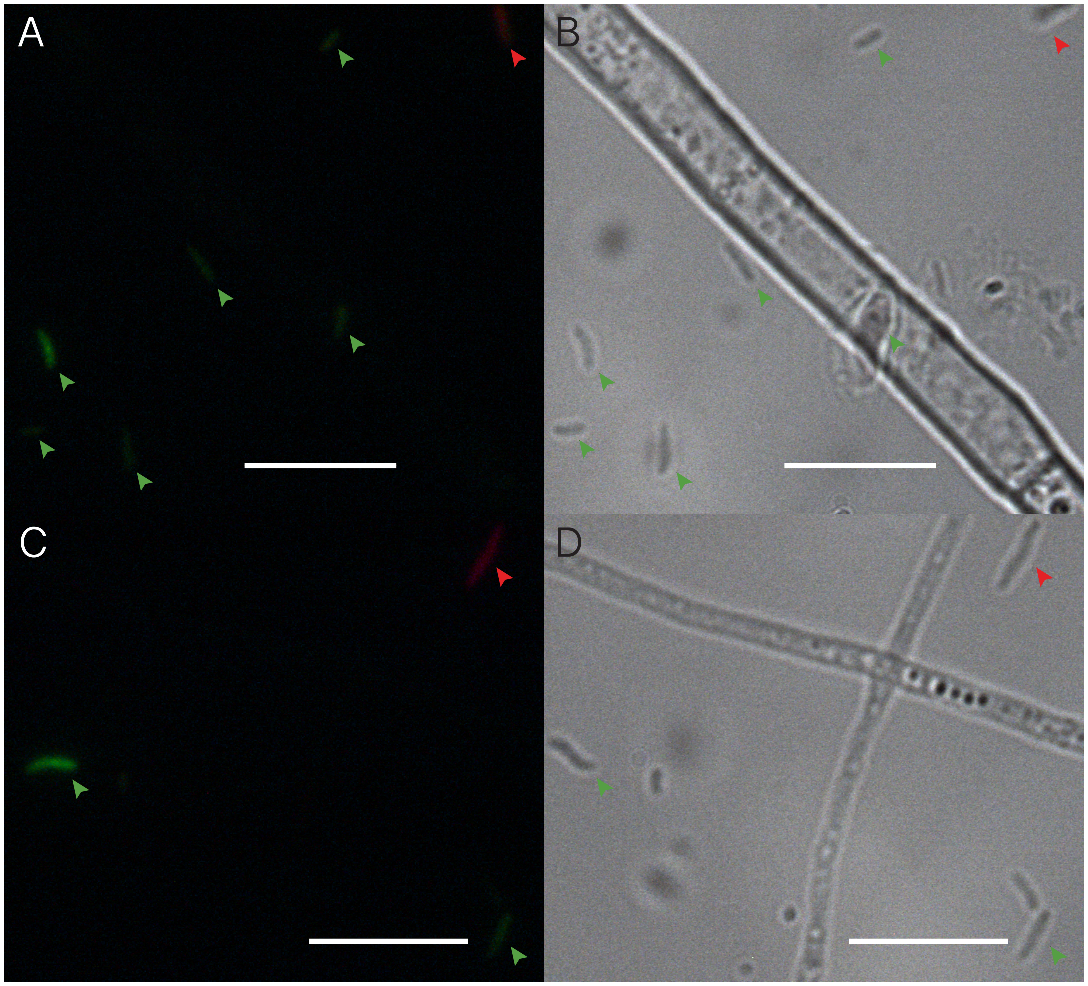

Supplementary Figure 1. Verification of successful visualization of extrahyphal bacteria, here viewed using Live/Dead staining. (A, C) Bacteria were introduced to axenic mycelia by spiking slide-mounting media with an aliquot of liquid bacterial culture. Fluorescently tagged, membrane-bound (i.e., viable) bacterial nucleic acids in green and those within compromised membranes (i.e., not viable) in red. **(B, D)** Same frames viewed with differential interference contrast (DIC). All scale bars = 10 µm. Contrast and brightness of images were enhanced 10% to improve visibility in the publication.

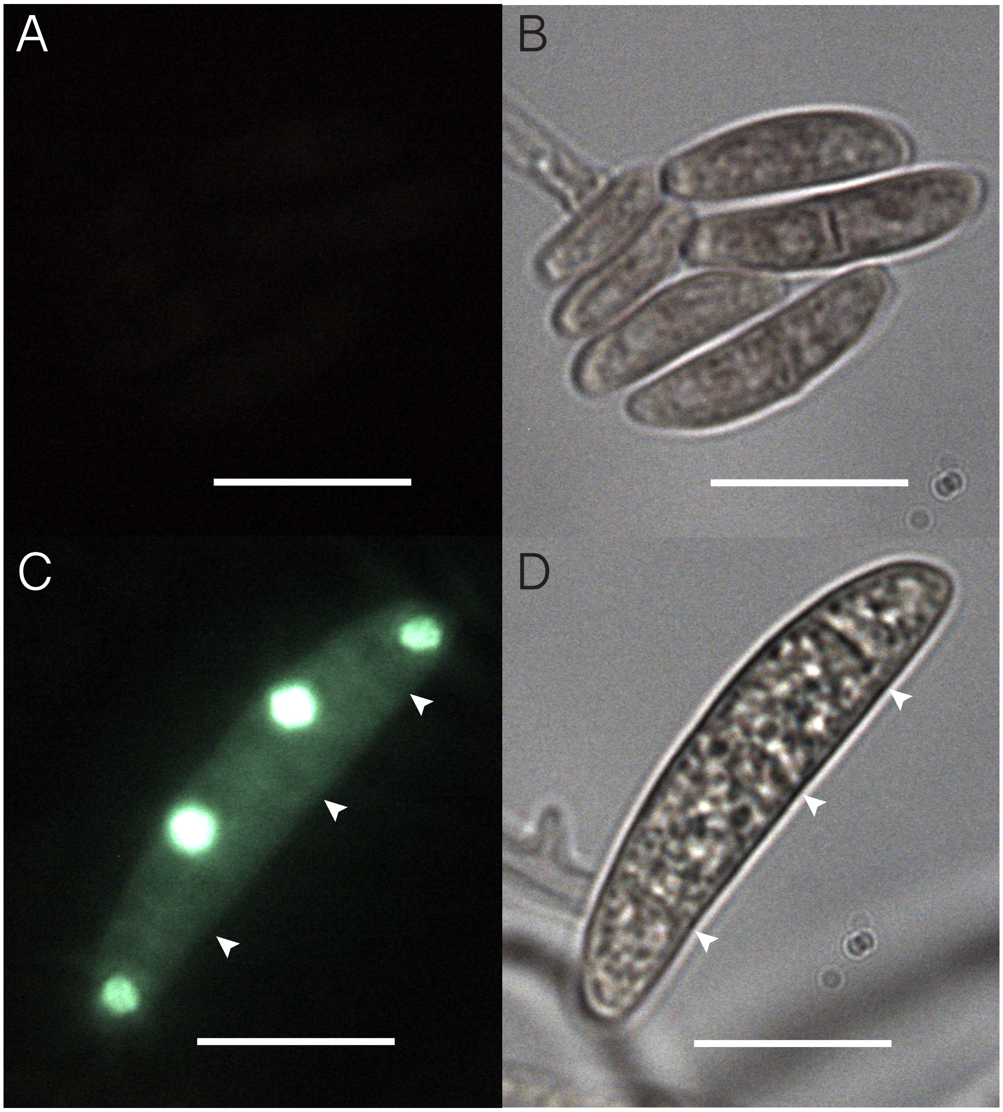

Supplementary Figure 2. Macroconidia of *Fusarium keratoplasticum* PS0362A viewed using Live/Dead staining show no evidence of EHB. (A) Conidiophore and developing macroconidia stained with Live/Dead show no evidence of EHB. **(B)** Same frame viewed with DIC. **(C)** A mature, multicellular macroconidium displaying single, viable fungal nuclei in each cell in green, but no evidence of EHB. **(D)** Same frame viewed with DIC. In (C) and (D), white arrows indicate septa. Photos are representative and no EHB were observed in spores of *F. keratoplasticum* PS0362A. Scale bars = 10µm.

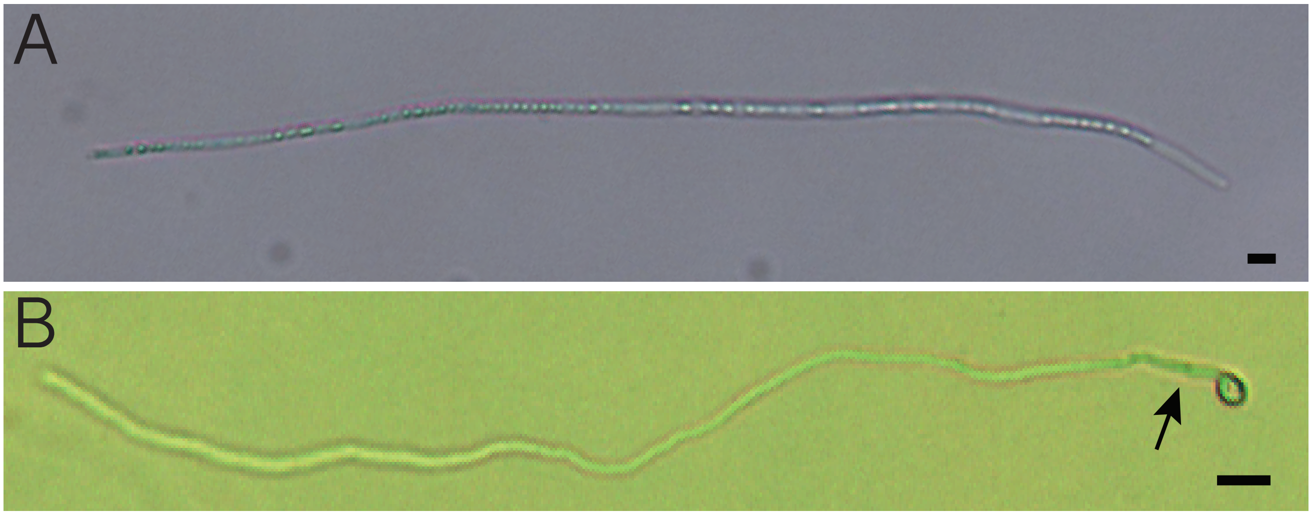

Supplementary Figure 3. Detail of hyphal suspensions used to inoculate phenotypic microarrays. (A) A hyphal fragment of representative length from suspensions used to inoculate phenotypic microarrays. Hyphal contents are visible, intact, and indicative of viability. Examination of hyphal suspensions following separation of spores revealed that few spores remained. Therefore, remaining spores were allowed to germinate for 6 h following preparation of suspensions in order to prevent metabolism associated with spore germination from affecting substrate use, and allow potential cross-infection of *Chitinophaga* sp. PS-EHB01 from blended mycelia into newly produced hyphae. **(B)** Confirmation that macroconidia remaining in the hyphal suspension did germinate before inoculation. Scale bars = 10μm.

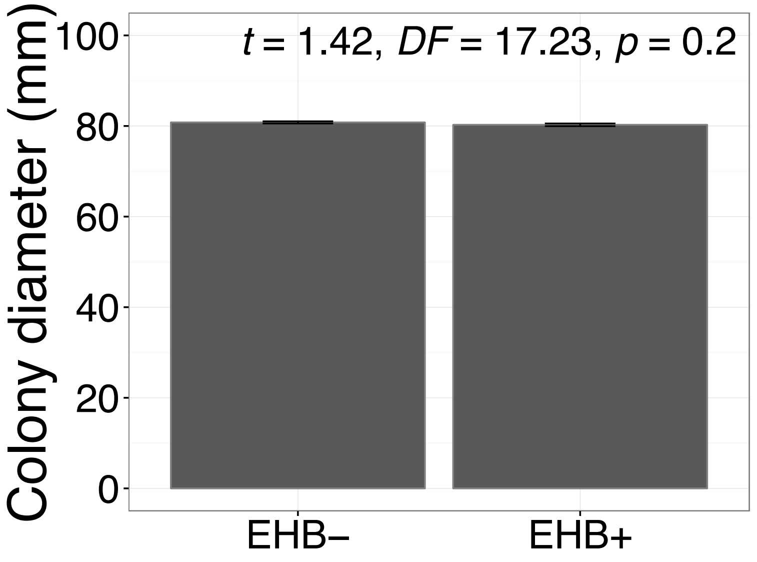

Supplementary Figure 4. Colony diameter of EHB+ and EHB– strains of *Fusarium keratoplasticum* PS0362A on a standard growth medium (2% MEA). Bars represent measurements from five replicate cultures of each strain. Results from a Welch *t*-test are shown.

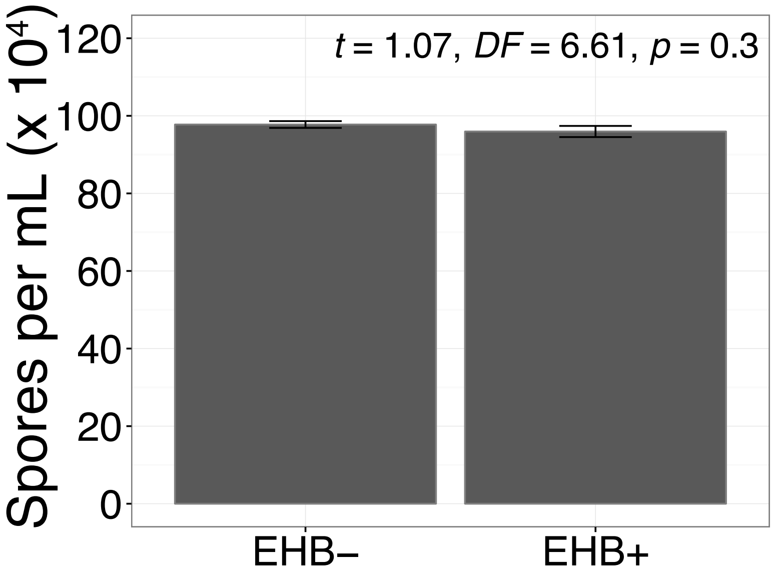

Supplementary Figure 5. Spore production compared between EHB+ and EHB– strains of *Fusarium keratoplasticum* PS0362A on a standard growth medium (2% MEA). Bars represent the number of conidia per mL from five replicate cultures of each strain. Results from a Welch *t*-test are shown.

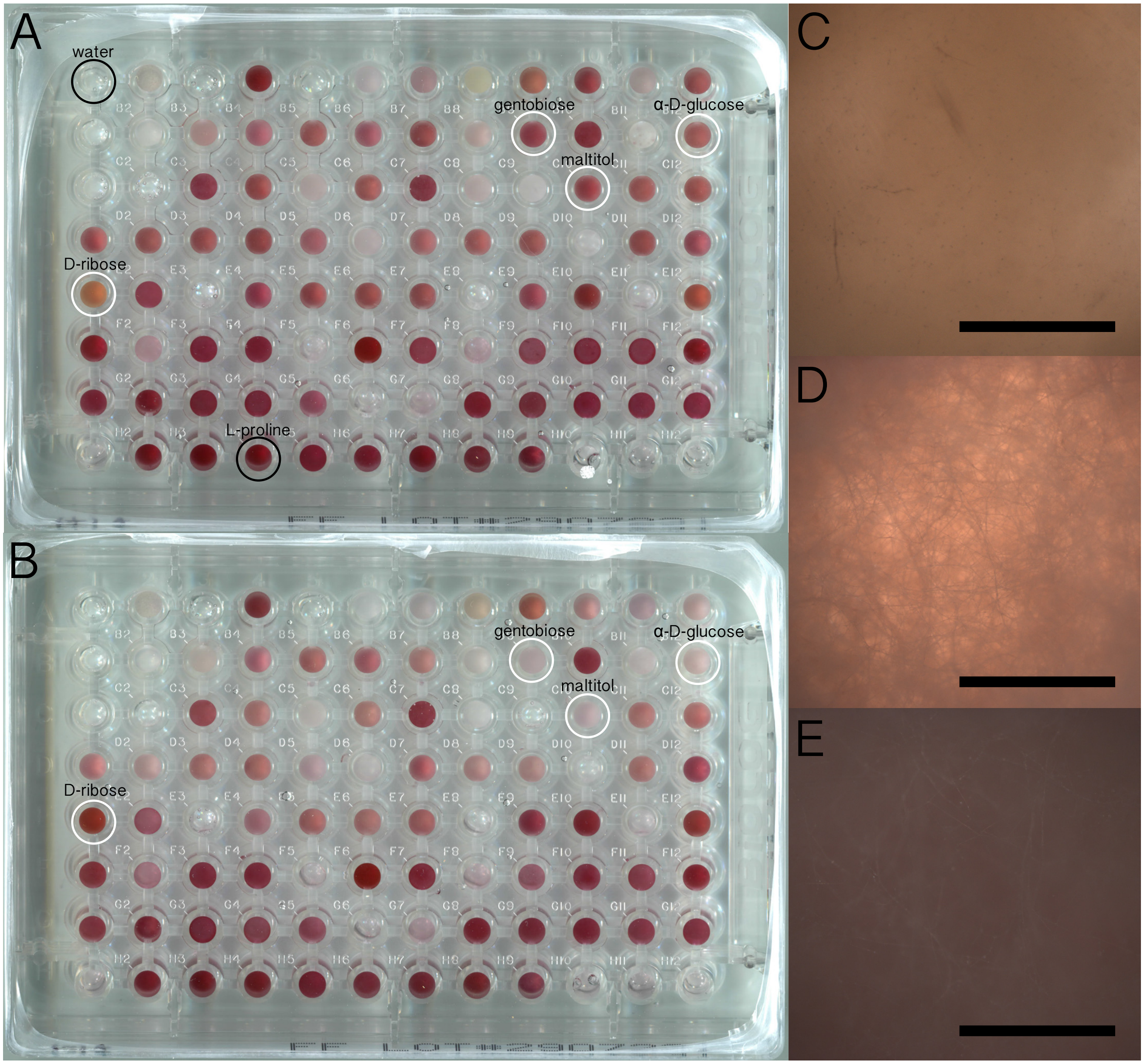

Supplementary Figure 6. Color change corresponding to cellular respiration across substrates in Biolog® Filamentous Fungus (FF) microplates at 7 d. (A) Plate illustrating respiration of EHB+ *Fusarium keratoplasticum*. Black circles highlight water and L-proline, which exemplify negligible growth ($A_{750}{}^{7} \leq 0.3$) and moderate growth, respectively. White circles highlight exemplar substrates for which qualitative differences between EHB+ and EHB– strains can be seen in the amount of color change corresponding to cellular respiration. **(B)** Plate illustrating respiration of EHB– *Fusarium keratoplasticum*. **(C)** Representative photomicrographs of hyphal density produced on water, **(D)** α-D-glucose, and **(E)** L-proline. Note the lack of growth and absence of color change in water, and that the light apparent through the hyphae on α-D-glucose is obscured by the more dense hyphae on L-proline. All micrographs are of the EHB+ strain with the same lighting. Scale bars = 500µm.

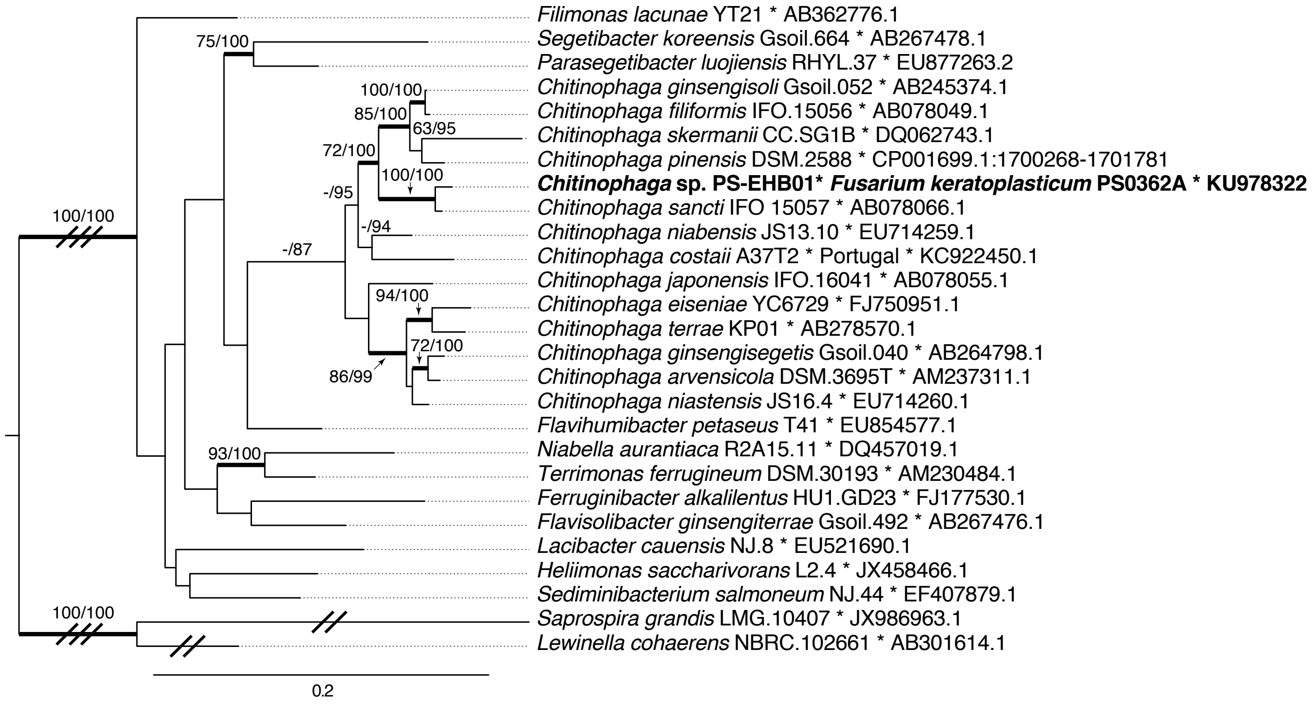

Supplementary Figure 7. Phylogenetic relationships within Chitinophagaceae (Bacteroidetes) based on 16*S* rRNA, showing the placement of the focal endohyphal bacterium, *Chitinophaga* sp. PS-EHB01. Results of maximum likelihood analysis with branch support values indicating maximum likelihood bootstrap (MLBS) and Bayesian posterior probabilities (BPP). Thickened branches are those with ≥70 MLBS and ≥95 BPP values. Branches lacking support values have values <60 MLBS and <80 BPP. Double- and triple hash marks indicate branches shortened to one-half and one-quarter of their length, respectively. The taxon label for *Chitinophaga* sp. PS-EHB01 is bolded. All taxon labels include GenBank accession numbers. The focal endohyphal bacterium is sister to *C. sancti* and closely related to *C. pinensis,* the only congener for which genomic data are currently available. The outgroup includes two members of the Saprospiraceae, the only other family in the Chitinophagales. Final treelength = 2.85; final optimized –ln(*L*) = –8767.91; Bayesian trees retained after burn-in: 4,366.

3.2 Supplementary Tables

Supplementary Table 1. List of substrates pre-loaded onto Biolog® Filamentous Fungus (FF) microplates[1]. The classification of substrates followed Garland and Mills (1991).

Carbon sources		
Monosaccharides	Sugar alcohols	Amino acid derivatives
α-D-glucose	D-arabitol	amygdalin
D-arabinose	D-mannitol	
D-fructose	D-sorbitol	Carboxylic acids
D-galactose	glycerol	2-keto-D-gluconic acid
D-mannose	*i*-erythritol	α-ketoglutaric acid

[1] www.biolog.com

D-psicose
D-ribose
D-tagatose
D-xylose
L-arabinose
L-fucose
L-sorbose

Disaccharides
α-D-lactose
D-cellobiose
D-melibiose
D-trehalose
gentiobiose
isomaltulose
lactulose
maltose
sucrose
turanose

Trisaccharides
D-melezitose
D-raffinose
maltotriose

Tetrasaccharides
stachyose

Oligosaccharides
dextrin

Cyclic oligosaccharides
α-cyclodextrin
β-cyclodextrin

Nucleosides
adenosine
uridine

Polysaccharides

myo-inositol
maltitol
ribitol
xylitol

Methyl sugars
L-rhamnose
methyl α-D-galactoside
methyl β-D-galactoside
methyl α-D-glucoside
methyl β-D-glucoside

Alcoholic beta-glucosides
salicin

Glycosides
arbutin

Misc. Carbohydrates
sedoheptulosan

Amino sugars
D-glucosamine
N-Acetyl-D-galactosamine
N-Acetyl-D-glucosamine
N-Acetyl-D-mannosamine

Amino acids
L-alanine
L-proline
L-serine
L-threonine
L-aspartic acid
L-glutamic acid
N-acetyl-L-glutamic acid
glycyl-L-glutamic acid
L-pyroglutamic acid
γ-aminobutyric acid
L-asparagine
L-phenylalanine

β-hydroxybutyric acid
D-glucuronic acid
D-galacturonic acid
D-gluconic acid
D-saccharic acid
D-malic acid
L-malic acid
L-lactic acid
γ-hydroxybutyric acid
fumaric acid
p-hydroxyphenylacetic acid
quinic acid
sebacic acid
succinic acid

Esters
D-lactic acid methyl ester
succinic acid monomethyl ester

Amides
alaninamide
glucuronamide
succinamic Acid
ethanolamine
putrescine

Phosphorylated chemicals
glucose-1-phosphate
adenosine-5'-monophosphate

Brominated chemicals
bromosuccinic acid

Surfactants
Tween® 80

glycogen	L-ornithine
	L-alanyl-glycine

References

Darriba D., Toboada G. L., Doallo R., and Posada D. (2012). jModelTest 2: more models, new heuristics and parallel computing. *Nat. Methods* 9, 772. doi: 10.1038/nmeth.2109

Edgar R. C. (2004). MUSCLE: multiple sequence alignment with high accuracy and high throughput. *Nucleic Acids Res.* 32, 1792-1797. doi: 10.1093/nar/gkh340

Garland J. L. and Mills A. L. (1991). Classification and characterization of heterotrophic microbial communities on the basis of patterns of community-level sole-carbon-source utilization. *Appl. Environ. Microbiol.* 57, 2351-2359.

Huelsenbeck J. P. and Ronquist F. (2001). MRBAYES: Bayesian inference of phylogenetic trees. *Bioinformatics* 17, 754-755. doi: 10.1093/bioinformatics/17.8.754

Maddison W. P., and Maddison D. R. (2009). Mesquite: a modular system for evolutionary analysis. Version 2.6. http://mesquiteproject.org/

Miller M. A., Pfeiffer W., and Schwartz T. (2010). Creating the CIPRES Science Gateway for inference of large phylogenetic trees. *Proceedings of the Gateway Computing Environments Workshop (GCE)*, 14 Nov. 2010, New Orleans, LA. 1-8.

Proença D. N., Nobre M. F., and Morais P. V. (2014). *Chitinophaga costaii* sp. nov., an endophyte of *Pinus pinaster*, and emended description of *Chitinophaga niabensis*. *Int. J. Syst. Evol. Microbiol.* 64, 1237-1243. doi: 10.1099/ijs.0.053454-0

Ronquist F. and Huelsenbeck J. P. (2003). MrBayes 3: Bayesian phylogenetic inference under mixed models. *Bioinformatics* 19, 1572-1574. doi: 10.1093/bioinformatics/btg180

Shaffer J. (2017). justinshaffer/Endohyphal_bacterium_alters_substrate_use_by_Fusarium_keratoplasticum: Second release of code for analyzing Biolog FF microplate data [Data set]. Zenodo. doi: 10.5281/zenodo.250931

Shaffer J. P., Sarmiento C., Zalamea P.-C., Gallery R. E., Davis A. S., Baltrus D. A., and Arnold A. E. (2016). Diversity, specificity, and phylogenetic relationships of endohyphal bacteria in fungi that inhabit tropical seeds and leaves. *Front. Ecol. Evol.* **4**:116. doi: 10.3389/fevo.2016.00116

Stamatakis A. (2014). RAxML version 8: A tool for phylogenetic analysis and post-analysis of large phylogenies. *Bioinformatics* 30, 1312-1313. doi: 10.1093/bioinformatics/btu033
